# Structural behavior of HPC columns reinforced with CFRP bars under axial loading

**DOI:** 10.1038/s41598-026-68421-w

**Published:** 2026-09-15

**Authors:** Saad M. Badr, Ali S. Shanour, Taha A. El-Sayed, Gamal E. Abdelaziz

**Affiliations:** Structural Engineering Department, Faculty of Engineering at Shoubra, Benha University, Cairo, Egypt

**Keywords:** RC columns, CFRP bars, Axial load, Tie spacing, Ultimate capacity, Theoretical results, Engineering, Materials science

## Abstract

This study investigates the axial compressive behavior of high-performance concrete (HPC) columns reinforced with carbon fiber-reinforced polymer (CFRP) bars, motivated by CFRP’s high strength, fatigue resistance, and corrosion immunity. Despite these advantages, limited research has constrained its practical use. Eight HPC columns (150 × 150 mm cross-section, 1200 mm height) made of M50-grade concrete with 8% silica fume and superplasticizer were tested under axial loading. Key parameters included axial load capacity, deformation, failure mode, and ductility. Results showed that CFRP-reinforced columns exhibited only a slight 5.7% reduction in ultimate load compared to steel-reinforced counterparts. However, CFRP specimens absorbed approximately 8% more energy while maintaining comparable ductility. No CFRP bar rupture occurred during testing; the maximum strain recorded in the longitudinal CFRP bars reached approximately 65% of their ultimate tensile strain capacity (2600 μm/m out of 4000 μm/m). Theoretical strength predictions using existing confined-concrete models aligned with experimental data. Overall, the study demonstrates that CFRP longitudinal bars and stirrups offer a viable alternative to conventional steel reinforcement in HPC columns. The minor reduction in load capacity is offset by improved energy absorption and environmental benefits, including corrosion resistance and potentially longer service life. These findings support broader application of CFRP in concrete structures, particularly where durability under aggressive conditions is critical. The work contributes valuable experimental data and validates analytical models, helping to bridge the knowledge gap hindering wider adoption of CFRP reinforcement in real-world construction.

## Introduction

High-Performance Concrete (HPC) has superior strength, ductility, and durability^1,2^. Reinforced concrete (RC) buildings rely on columns to transmit loads from upper to lower levels and then release them into footings. Column failure can be disastrous in a structure^3,4^. Corrosion of reinforcing steel is a concern in traditional reinforced concrete (RC), especially in hostile locations^5,6^. In certain structures, such as magnetic resonance imaging and other radiation units, steel should not be present around an MRI device. Fortunately, fiber-reinforced polymer (FRP) provides a great alternative in these circumstances, owing to its non-corrosive, non-magnetic, and non-conductive properties^7,8^.

Previous studies on several types of FRPs deemed Carbon-FRP (CFRP) to be the least prone to creep and fatigue rupture^9–11^. Furthermore, various studies have proved CFRP’s efficiency as a key reinforcing material in a range of structural elements^12–14^. Thus, CFRP bars are effective alternatives for steel bars, owing to minor variations in elastic modulus; unlike steel, CFRP is highly flexible and can be fabricated into more complex shapes, giving a cost-effective structural solution^4,8,15–19^.

Moreover, CFRP is significantly lighter than steel and can be easily cut to the required length on-site, making it a versatile material for construction applications^11,19,20^. Despite the growing interest in CFRP applications, the behavior of CFRP-reinforced concrete (CFRP-RC) columns remains relatively under-explored^13,21,22^. It has been reported that CFRP bars have a compressive strength of about 78% of their tensile strength^5,23^.

Recent research has investigated the stress-strain behavior of high-strength concrete confined with CFRP in elements with circular and square cross-sections. These studies revealed that CFRP confinement is more effective in circular specimens, leading to enhanced performance and stated that it offered enough restriction against buckling of the longitudinal FRP bars and satisfactory confinement of the concrete core in the post-peak periods^4,5,24,25^.

Previous studies on concrete columns internally reinforced with CFRP bars have primarily focused on normal-strength concrete (NSC). Afifi et al.^13,26^, investigated the axial capacity of circular concrete columns reinforced with CFRP bars and spirals, reporting that CFRP bars can contribute effectively to axial capacity when adequate confinement is provided. Hadhood et al.^15,21^, studied circular HSC columns reinforced with CFRP bars under axial and eccentric loads and proposed design equations. Tobbi et al.^22,27^ examined concrete columns reinforced longitudinally and transversally with FRP bars and concluded that FRP bars can serve as viable reinforcement in compression members. Mohamed et al.^14^, evaluated the performance of concrete columns with FRP bars and hoops/spirals under axial loading. More recently, El-Sayed et al.^5^, studied UHPC columns reinforced with basalt bars, and Abed et al.^28^, investigated the effect of BFRP ties on the axial performance of RC columns. However, limited research exists on HPC columns with CFRP longitudinal and transverse reinforcement, particularly regarding the interaction between HPC’s brittle nature and CFRP’s linear-elastic behavior under concentric axial loading.

Kamiński and Trapko^29^, introduced a novel strengthening approach for structural CFRP elements in reinforced concrete members, wherein the CFRP was applied selectively in designated areas. Berthet et al.^30^, analyzed the influence of E-glass and carbon CFRP on the behavior of five classes of concrete strength, ranging from 24 to 170 MPa. Park et al. (2008) utilized narrow CFRP strips in various configurations to reinforce concrete columns with compressive strengths of 20.72 and 26.08 MPa. Zaki et al. (2019) proposed the use of CFRP fiber anchors and dowels as reinforcement for T-shaped beams^31,32^.

High-performance concrete (HPC) was selected for this study because it is widely used in heavily loaded structural members, such as high-rise buildings, bridge piers, and marine infrastructure, where high strength, durability, and reduced member dimensions are desirable. In addition, the corrosion resistance of CFRP reinforcement makes it particularly suitable for such aggressive service environments. Compared with normal-strength concrete (NSC), HPC exhibits higher compressive strength but a more brittle post-peak response, making effective confinement more critical. In contrast, UHPC generally requires substantially higher confinement pressures due to its much higher compressive strength. Therefore, HPC with a compressive strength of approximately 50 MPa represents a practical intermediate material for evaluating the performance of CFRP-reinforced columns.

To address the aforementioned research gap, the current research presents an alternative reinforcement method for HPC columns using CFRP bars, which may enhance axial load capacity while providing environmental benefits compared to conventional steel-reinforced RC columns. To this end, experimental work was conducted on eight HPC column specimens [four reinforced with steel and four reinforced with CFRP] subjected to axial loading. In addition, a theoretical study was conducted to predict the ultimate load-carrying capacity of all HPC column specimens. The detailed investigation is described as follows:

- Analysis of the structural properties of carbon-reinforced columns (longitudinal and stirrups) to determine their failure mechanism.
- Evaluating the impact of axial load from carbon bars on concrete columns.
- Conducting a theoretical study on the studying high-performance concrete (HPC) columns.
- Comparison of theoretical results with experimental findings to predict axial stress in the column.

## Experimental study

### Materials

#### Cement

In this study, Ordinary Portland Cement (OPC) CEM I 42.5 N conforming to the Egyptian Standard Specification ESS 203/2020^33^, was used. The cement was partially replaced with 8% silica fume by weight to produce blended cement. Both physical and chemical properties of the cement are consistent with the Egyptian Standard Specifications as shown in Tables 1 and 2.

**Table 1:** Chemical analysis of the reference cement CEM I 42.5 N.

Chemical component	Chemical composition (%)	ESS limits
SiO_2_	19.432	- - -
Fe_2_O_3_	3.982	- - -
Al_2_O_3_	5.778	- - -
CaO	66.403	- - -
MgO	1.710	- - -
SO_3_	2.632	Not more than 3.5%
Na_2_O	0.432	- - -
K_2_O	0.233	- - -
Loss on ignition	2.791	Not more than 5%
Insoluble residue	0.567	Not more than 5%

**Table 2:** The physical properties of the reference CEM I 42.5 N.

Properties	Values		ESS limits
Setting Time	Hour	Min	
Initial	1	40	Not less than 60 min
Specific surface area	3610 cm^2^/g		
Compressive Strength (kg/cm^2^)			
2-days	298		Not less than 100 kg/cm^2^
28-days	435		Not less than 425 kg/cm^2^

#### Aggregates

**Fig. 1:**
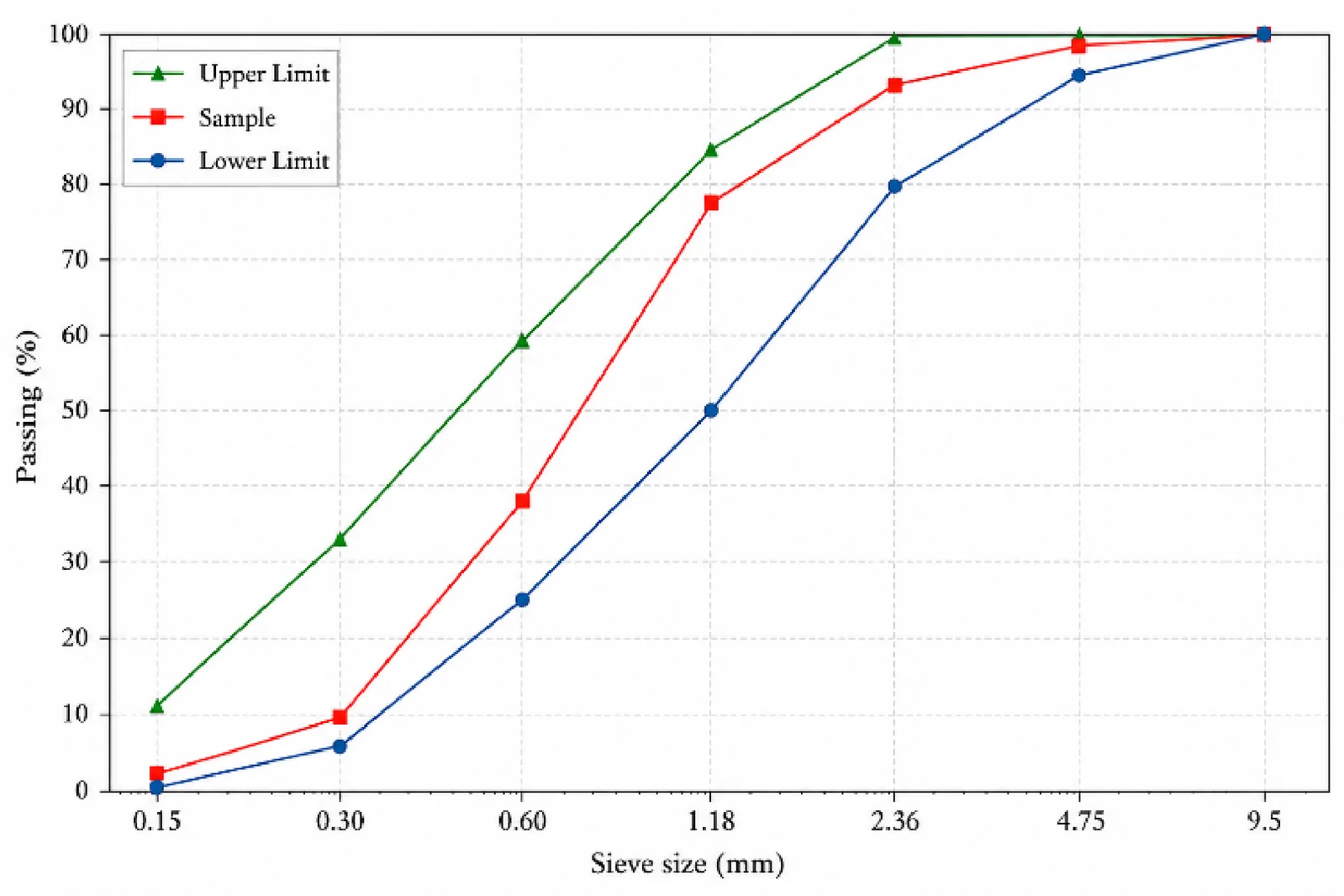
Sieve analysis of the fine aggregates.

The fine aggregates used were natural sand. Its physical properties are given in Table 3, and Fig. 1 shows the grading of the sand. the results indicate that the fine aggregates comply with the standard specifications ESS 203/2020^33^.

**Table 3:** The physical properties of the fine aggregates (sand).

Sieve analysis of (natural sand)									
Sieve size (mm)	9.5	4.75	2.36		1.18	0.6		0.3	0.15
Passing %	100.0	98.7	95.2		79.1	37.6		9.2	1.3
Lower Limit	100.0	100.0	100.0		85.0	60.0		30.0	10.0
Upper Limit	100	95.0	80.0		50.0	25.0		5.0	0.0

Properties				Value			ESS limits		
CL^−^ %				0.039			Not more than 0.06%		
SO_3_ %				0.095			Not more than 0.4%		
Clay and Fine content (% by volume)				1.12			Not more than 3.0%		
Specific gravity (t/m^3^)				2.63			2.5–2.75		
Dry Unit Weight (t/m^3^)				1.62			- - -		
Water Absorption Ratio, %				0.21			Not more than 2.0%		

**Fig. 2:**
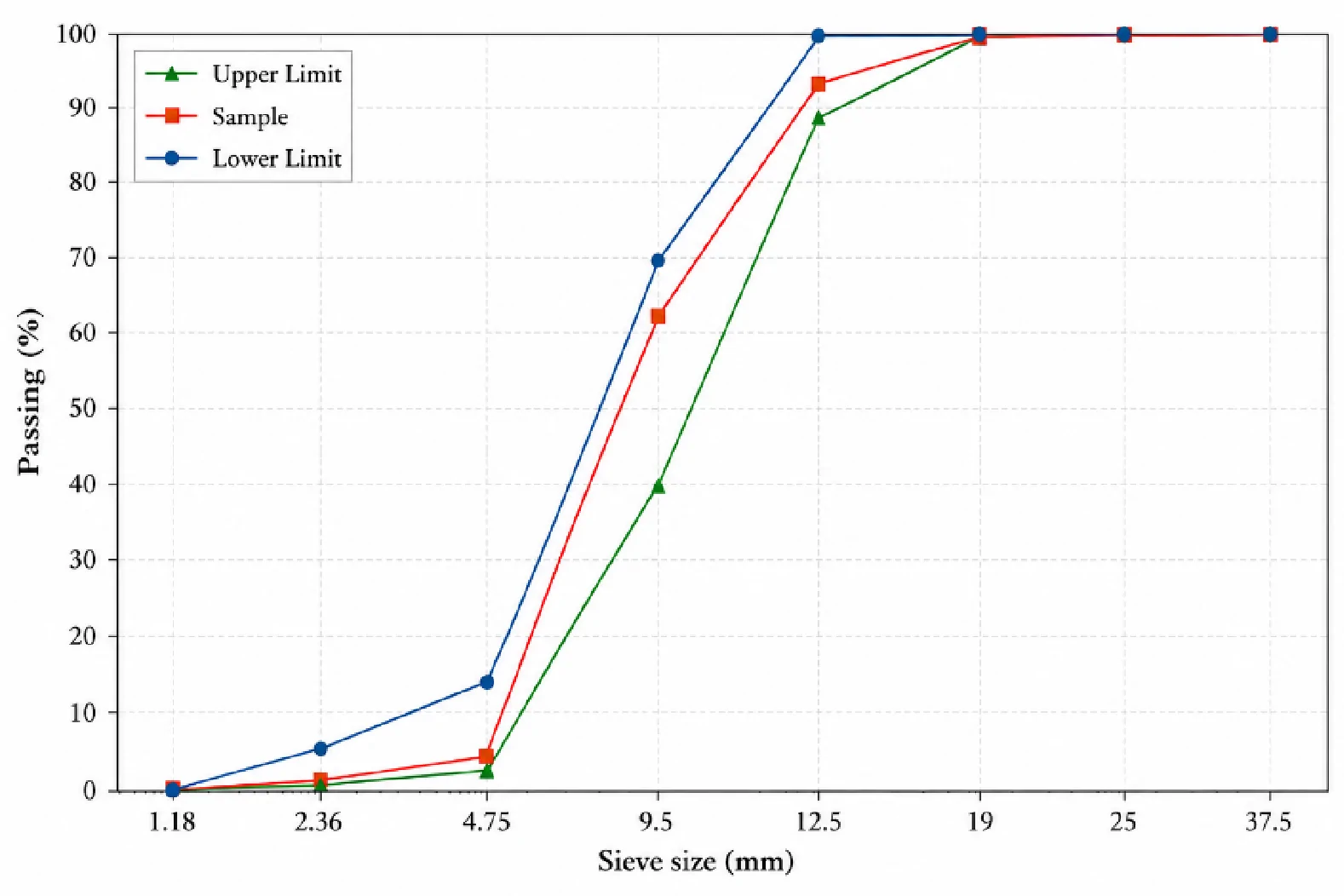
Sieve analysis of the coarse aggregates (dolomite size#1).

Crushed dolomite from the Ataka quarry in Suez Governorate was used as coarse aggregate. The maximum size retained on the 4.75 mm sieve was 12.5 mm. Its physical properties are given in Table 4, and Fig. 2 shows the grading of the aggregates. The results indicate that the coarse aggregates comply with the standard specifications ESS 203/2020^33^.

**Table 4:** The physical properties of the coarse aggregates (dolomite size#1).

Sieve analysis of (dolomite size#1)										
Sieve size (mm)	37.5	25	19	12.5		9.5	4.75		2.36	1.18
Passing %	100.0	100.0	100.0	94.5		63.8	3.1		1.4	0.0
Lower Limit	100.0	100.0	100.0	100.0		70.0	15.0		5.0	0.0
Upper Limit	100.0	100.0	100.0	90.0		40.0	0.0		0.0	0.0

Main characteristics of (dolomite size#1)										
Properties					**Value**			**ESS limits**		
Max. Nominal Size (mm)					12.5			--------------------		
CL^−^ %					0.024			Not more than 0.04%		
SO_3_ %					0.035			Not more than 0.4%		
Specific gravity (t/m^3^)					2.64			2.5–2.75		
Dry Unit Weight (t/m^3^)					1.68			------------------		
Water Absorption Ratio, %					0.9			Not more than 2.5%		

#### Silica fume (SF)

Silica fume was used as a partial replacement for cement at a level of 8% by weight to maximize the improvement in mortar strength. In this study, Elkem Micro Silica^®^ Grade 920 was obtained from a local supplier with Chemical analysis as shown in Table 5. The surface area of Silica fume was 20,960 (cm²/g). It was a very fine powder with a characteristic grayish color.

Previous research’ findings informed the selection of the replacement ratio^2,34^. Silica fume is mostly made up of silicon dioxide (SiO₂), which accounts for 92–94% of its composition. This high silica content accounts for its significant pozzolanic activity, which allows for an effective reaction with cement hydration products while also increasing the mortar’s strength and durability. In addition to silica, silica fume contains a minor amount of carbon (3–5%), which may have a slight impact on its color and performance.

Trace amounts of other oxides, including iron oxide, calcium oxide, aluminum oxide, magnesium oxide, as well as potassium, sodium, and manganese oxides, are also present. Although these constituents occur in very small quantities, they can have a limited yet occasionally beneficial effect on the chemical reactions in cementitious systems.

**Table 5:** Chemical analysis of Silica fume sample.

Chemical component	Chemical composition (%)
SiO2	95.52
Fe2O3	0.66
Al2O3	0.23
CaO	0.76
MgO	0.40
SO3	0.46
Na2O	0.23
K2O	0.71
Loss on ignition	0.72

#### Superplasticizer (SP)

The liquid polycarboxylate superplasticizer with a density of 1085 kg/m³ and a chloride content of less than 0.2% was supplied by BASF Egypt (MasterGlenium^®^ 51). The admixture conforms to the Egyptian Standard Specification (ESS 203/2020) for chemical admixtures in concrete and to ASTM C494 Type F^33^. At the adopted dosage of 1.5% by weight of cementitious materials, the superplasticizer provides a water-reducing capacity of approximately 30%.

#### Steel bars

For Group A columns, 12 mm high-tensile deformed bars conforming to Egyptian Standard Specification ESS 203/2020 (Grade 42/60) were used as longitudinal reinforcement. For stirrups, 6 mm and 8-mm mild steel round bars conforming to Egyptian Standard Specification ESS 203/2020^33^, (Grade 24/35) were used.

The steel bars were tested in accordance with ASTM A370. The measured properties were:

- 12 mm deformed bars: fy = 435 MPa, fu = 612 MPa.
- 6 mm mild bars: fy = 315 MPa, fu = 442 MPa.
- 8 mm mild bars: fy = 328 MPa, fu = 456 MPa.

#### CFRP bars

**Fig. 3:**
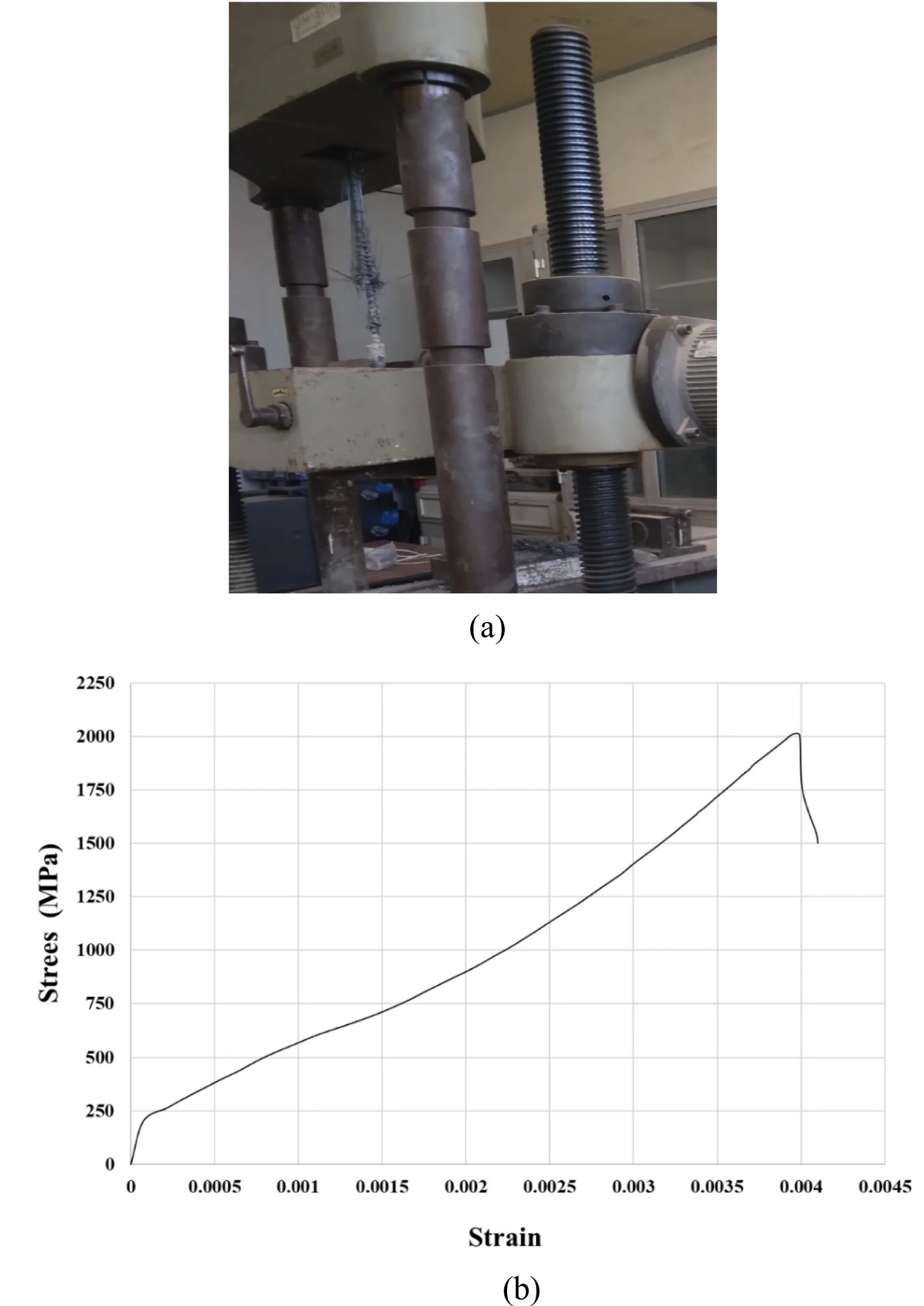
CFRP bars used in the experimental program; (**a**) tensile failure mode of the CFRP bar after testing; (**b**) Observed Carbon Fiber Bar Stress - Strain Curve for φ 11.2 mm.

Deformed high tensile Carbon fiber bars of 12 mm diameter were prepared locally. The characteristics of carbon bars with tensile strength ~ 2013 MPa where Tensile strength equal (198200/(π*11.2²)/4)) = 2013 MPa) as per Fig. 3 and Tensile modulus of elasticity ~ 111.3 GPa and ultimate strain (ε_u_) ~ 0.4%, that it is comply with^18,20,35,36^.

The compressive properties of the CFRP bars were determined through compression testing in accordance with ASTM D695. The measured values were:

- Compressive strength: 1540 MPa (approximately 76.5% of the tensile strength of 2013 MPa).
- Compressive modulus of elasticity: 92.5 GPa (approximately 83% of the tensile modulus of 111.3 GPa).
- Compressive strain at failure: approximately 0.32%.

It should be noted that the strain was calculated from the crosshead displacement of the testing machine. Therefore, the initial portion of the stress–strain curve includes the effects of machine compliance and grip seating rather than the intrinsic behavior of the CFRP bar, which remained essentially linear elastic until brittle failure.

**Fig. 4:**
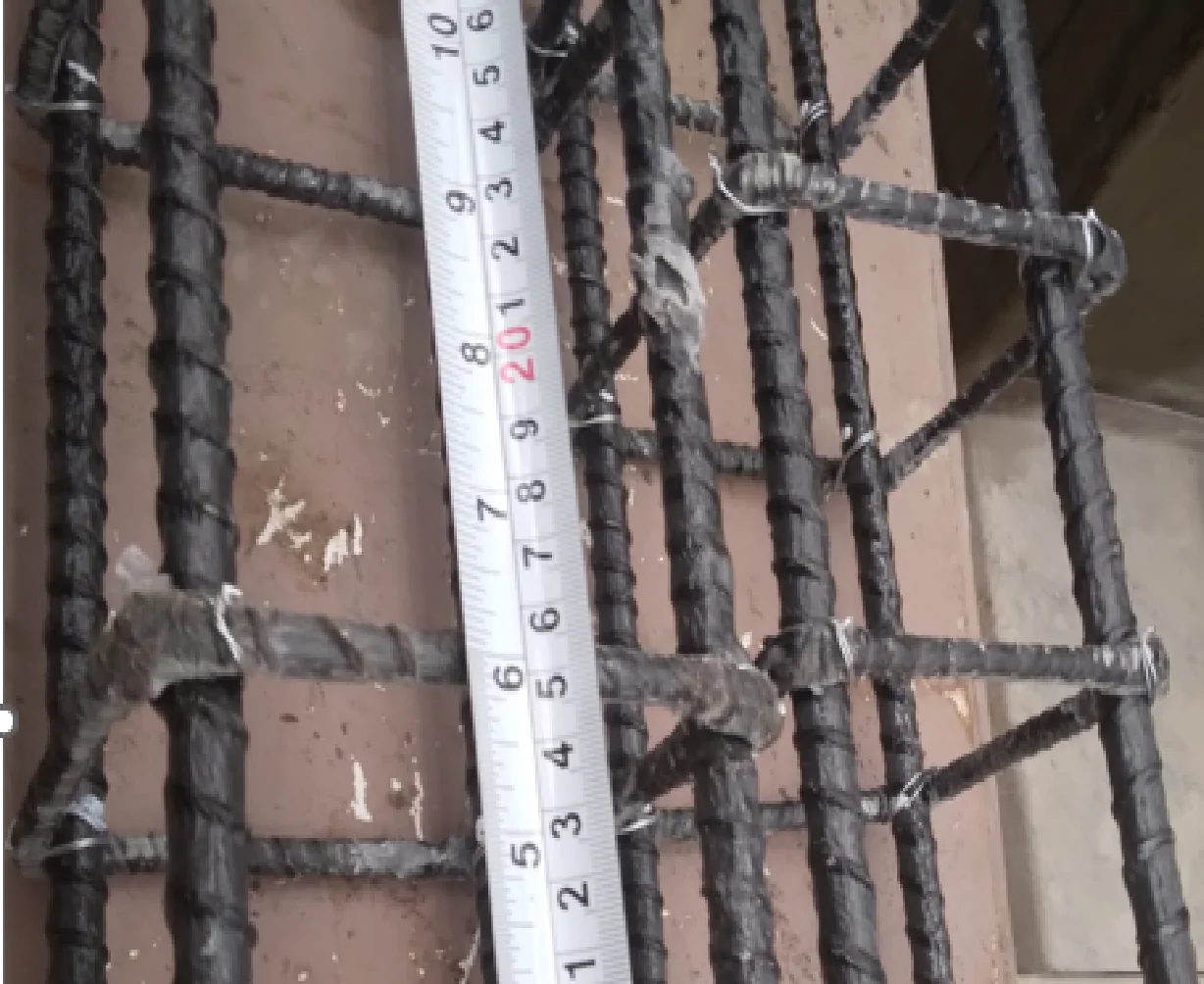
Manufacturing closed-loop carbon fiber reinforced polymer (CFRP) straps using the manual method.

Closed CFRP stirrups with a square cross-section of 100 × 100 mm were manually fabricated using a wet lay-up filament winding technique as shown in Fig. 4. High-strength carbon fiber tows were impregnated with a two-component epoxy resin and wound around a rigid square mold coated with a wax-based release agent.

The fibers were laid in successive layers with a uniform pitch of ~ 7 mm, applying light pressure to ensure consolidation and minimize voids. Stirrup diameters of 6 mm and 8 mm were achieved by controlling the number of fiber layers and winding density, with excess resin removed to optimize the fiber-to-resin ratio. The stirrups were cured under ambient conditions according to the epoxy manufacturer’s guidelines, with optional post-curing to enhance crosslinking and mechanical properties. After curing, the stirrups were demolded and finished to remove surface irregularities. Manual wet lay-up was chosen due to its flexibility in producing complex closed-loop geometries and precise fiber orientation, particularly at bends and corners where stress concentrations are high. This approach enables small-batch production while preserving high fiber volume fraction and minimizing resin-rich zones, resulting in optimal performance as concrete reinforcement.

### Mix design

The typical compressive strength of the design combination was estimated to be 50 MPa. Casting was done according to Egyptian Standard Specification ECP 203/2007^37^, for concrete mix design. Table 6 lists the properties of the mixture.

**Table 6:** Mix design.

Item	Cement kg/m^3^	Silica Fume (SF) kg/m^3^	Water kg/m^3^	Water-Cement W/C ratio	Water-binder W/b ratio	Dolomite (kg/m^3^)	Sand (kg/m^3^)	SP (L/m^3^)
Per m^3^ of HPC	575	50	138	0.24	0.22	1100	580	18

Because of developments in concrete technology, high-performance concrete is now widely available and increasingly used. Concerns have been raised about the effectiveness of HPC columns, as the use of HPC to reduce cross-sectional dimensions favors the building of RC columns over conventional strength concrete^5^.

The mixing procedure followed this sequence: (1) coarse and fine aggregates were dry-mixed for 2 min; (2) cement and silica fume were added and mixed for 1 min; (3) half of the mixing water (with SP) was added and mixed for 2 min; (4) the remaining water (with SP) was added and mixed for an additional 3 min. Total mixing time was 8 min using a mechanical rotary mixer. The concrete was cast into steel molds in three layers, each compacted using a vibrating Table (50 Hz) for 15 s. After 24 h, specimens were demolded and cured under controlled conditions at 22 ± 2 °C and > 95% relative humidity for 56 days.

**Fig. 5:**
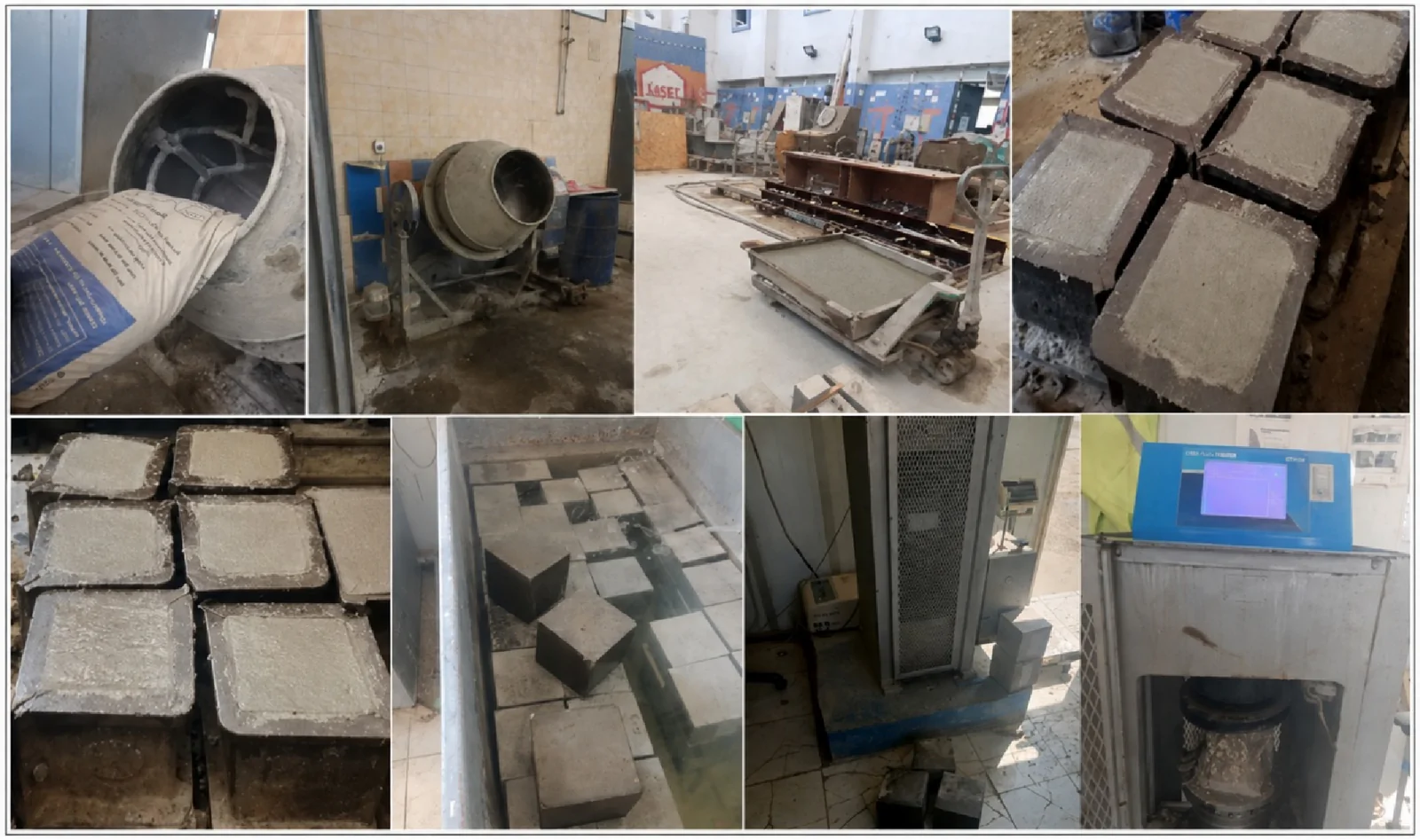
Mechanical rotary mixer and cubes curing and testing by Compressive strength testing machine.

The workability of the HPC mixture was assessed using slump flow and T50 measurements in accordance with ASTM C1437: slump flow = 680 ± 10 mm, T50 time = 3.2 ± 0.3 s. The reported concrete compressive strength values represent the average results of the three specimens at 7, 28 and 56 days as per Table 7; Fig. 5.

**Table 7:** Compressive strength results.

Cube no	At 7 days			At 28days			At 56 days		
	Weight kg	Density kg/m^3^	F_cu_ (MPa)	Weight kg	Density kg/m3	F_cu_ (MPa)	Weight kg	Density kg/m3	F_cu_ (MPa)
1	8.06	2388	40.8	8.09	2397	49.3	8.11	2403	52.4
2	8.11	2403	39.7	8.13	2409	48.8	8.16	2418	50.2
3	8.02	2376	40.1	8.05	2385	49.6	8.08	2394	53.7

### Experimental program and methodology

**Fig. 6:**
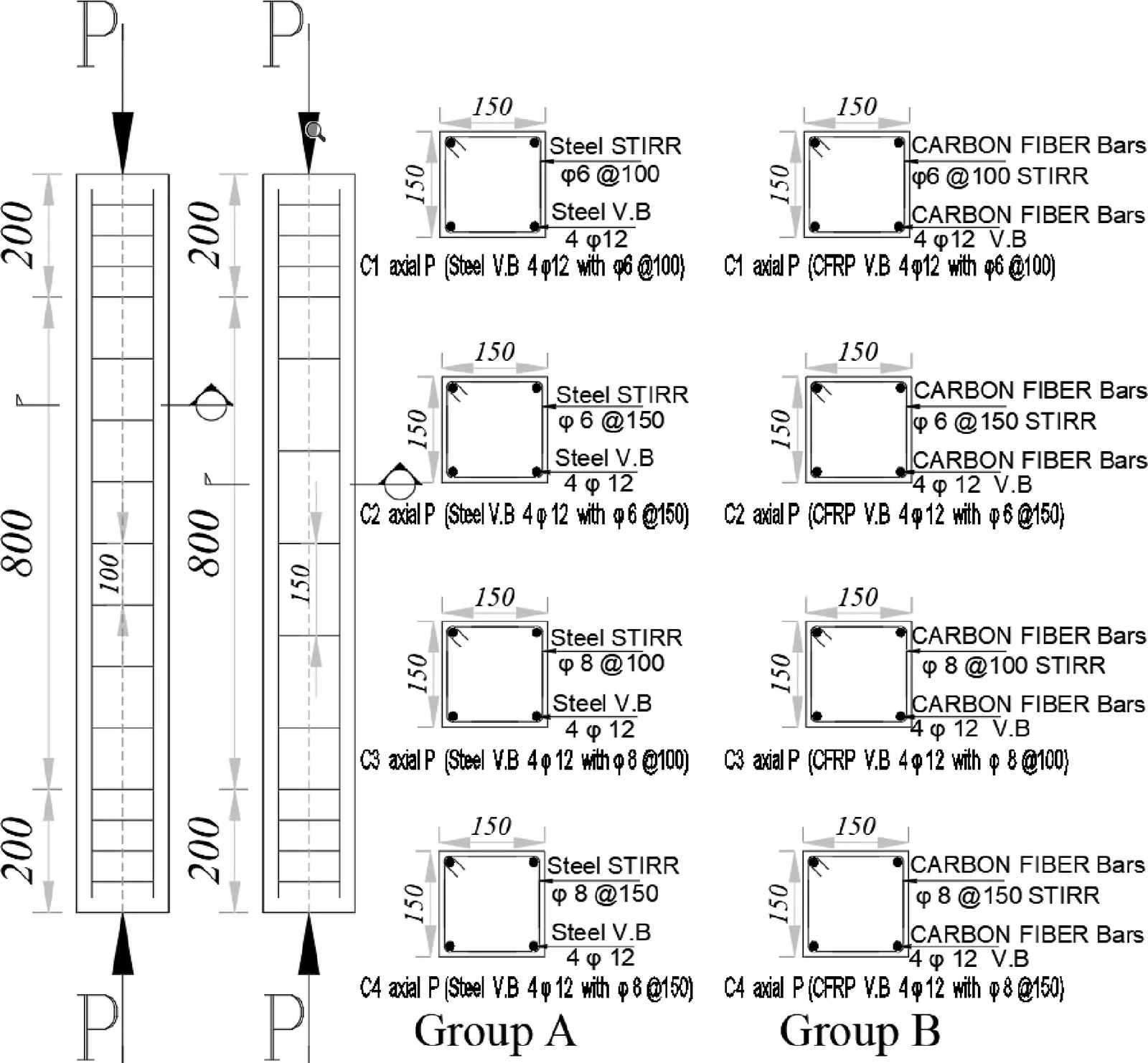
Concrete Columns typical dimensions and internal reinforcement details. “Group A consisted of steel longitudinal bars and steel ties, while Group B consisted of CFRP longitudinal bars and CFRP ties. Four 12 mm longitudinal bars were used in all specimens, whereas the transverse reinforcement consisted of 6 or 8 mm ties at spacings of 100 or 150 mm.”.

Table 8 presents the test matrix adopted in this study and Fig. 6 presents the data for eight columns that were used to examine the HPC columns’ overall behavior, failure mode, and final maximum capacity. All HPC columns had a square cross section with dimensions of 150 mm × 150 mm, and a height of 1200 mm. The actual compressive strength was determined based on the average 50.2 MPa.

**Table 8:** Specimen’s description.

Groups	Column ID	RFT. Type	Long. RFT	Trans. RFT
Group A	C1	Steel bars for Long. & Trans. RFT	4φ12	φ6@100 steel stirrups
	C2		4φ12	φ6@150 steel stirrups
	C3		4φ12	φ8@100 steel stirrups
	C4		4φ12	φ8@150 steel stirrups
Group B	C1	Carbon fiber bars for Long. & Trans. RFT	4φ12	φ6@100 carbon fiber stirrups
	C2		4φ12	φ6@150 carbon fiber stirrups
	C3		4φ12	φ8@100 carbon fiber stirrups
	C4		4φ12	φ8@150 carbon fiber stirrups

*RFT* Reinforcement.

The experimental program consisted of two groups (A&B), each including four specimens (C1, C2, C3 and C4), group (A); columns were reinforced with 12 mm steel bars as longitudinal reinforcement, with steel stirrups of 6 mm and 8 mm diameters spaced at 100 and 150 mm, while group (B) columns were reinforced with 12 mm CFRP bars as longitudinal reinforcement, with the same CFRP stirrup diameters and spacing as in group (A).

### Test setup

All columns were tested under displacement-controlled loading at a rate of 0.25 mm/min using a 5000 kN-capacity MTS hydraulic testing machine at the Housing and Building Research Center, where a versatile loading system is designed to evaluate structural components. Prior to formal testing, each column was preloaded to approximately 10% of the estimated ultimate load (≈ 80–100 kN) to verify load alignment, seating, and proper functioning of all instrumentation.

The column was then unloaded to zero before commencing the actual loading regime. Strain gauges were carefully positioned to capture key responses during loading: one gauge was mounted on a stirrup at the mid-height of the column (SG-S), and one gauge was placed on a longitudinal bar to record bar strains (SG-L). Also, another gauge was affixed to the concrete surface at the column’s mid-height to monitor concrete strain (SG-C).

**Fig. 7:**
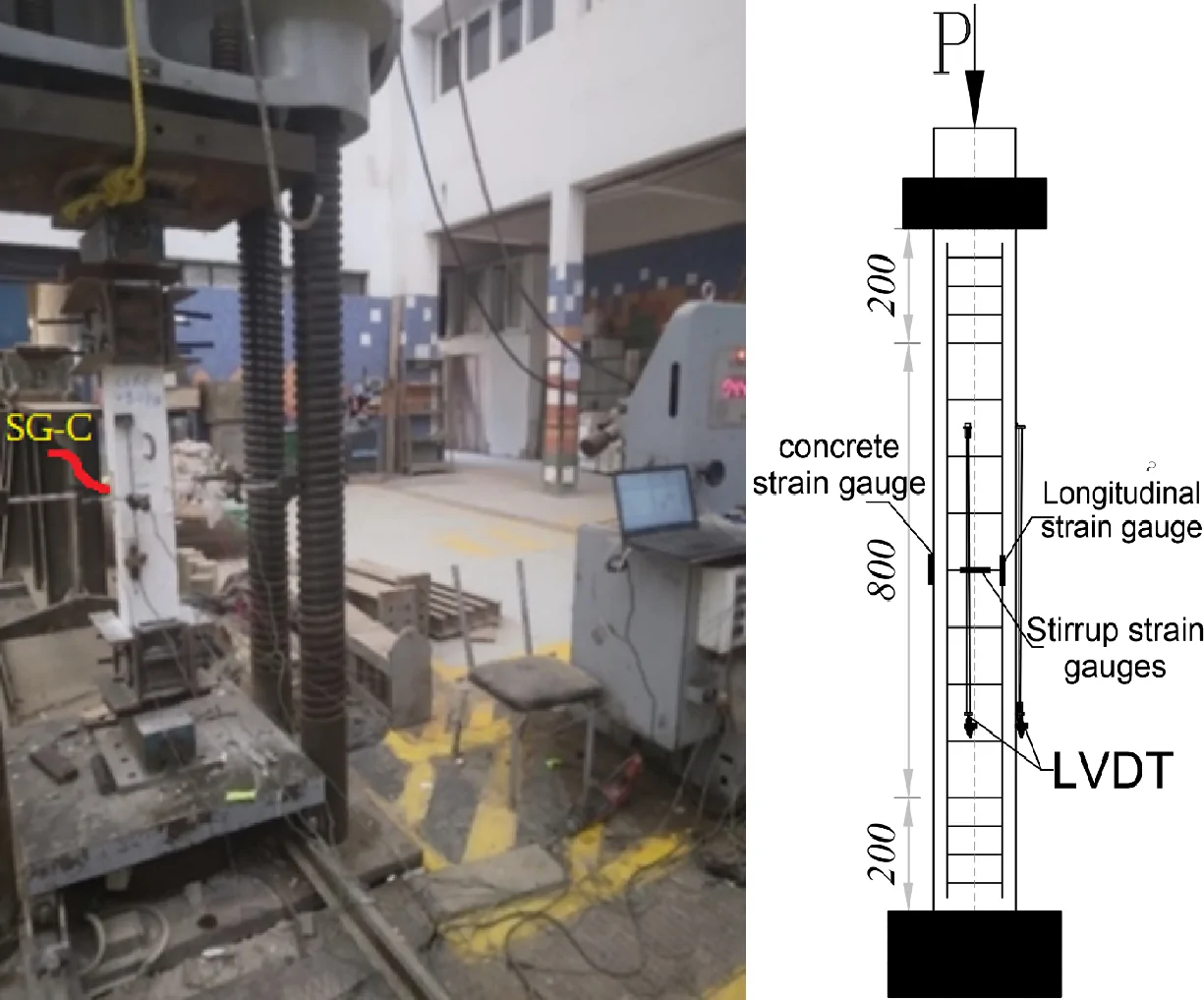
Experimental test setup for axial loading and testing of setup details with LVDTS and strain gauge locations of tested HPC column specimens.

**Fig. 8:**
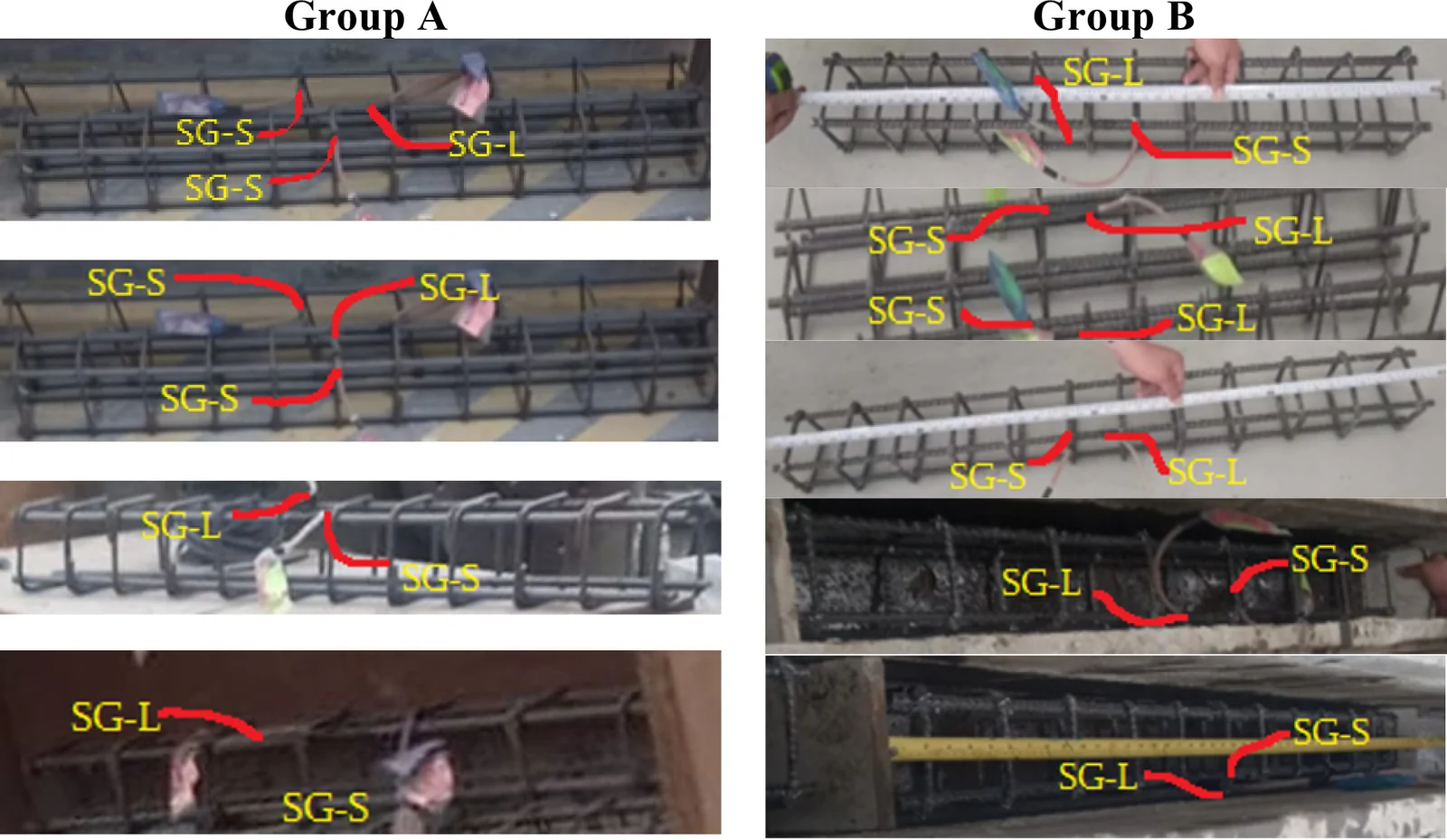
Overview of the assembled CFRP cages with Strain gauges.

Instrumentation specifications: LVDTs (Novotechnik TR-100, range ± 50 mm, accuracy ± 0.02% FS, 0.01 mm); strain gauges (Tokyo Sokki Kenkyujo, Type PFL-10–11, gauge length 10 mm, resistance 120 Ω, accuracy ± 0.5%); data acquisition (National Instruments cDAQ-9174, sampling frequency 10 Hz). Figures 7 and 8 present the overall test setup. The deformation behavior of each column was continuously tracked using linear variable differential transducers (LVDTs) from the start of loading until failure. Two loading heads made with high-strength steel plates were utilized to exert axial load on the column. The loading head conveyed the load from the MTS machine to the column via the ball joint, creating a hinged boundary condition. The loading-head parts were fastened together with high-strength bolts.

## Experimental results and discussion

### The cracking load and ultimate load behavior

The first cracking load (P_cr_) was identified through visual observation of the concrete surface during testing, complemented by monitoring strain gauge readings on the concrete surface at mid-height; a sudden change in the slope of the load-strain curve indicated crack initiation. According to Table 9, the first cracking load was consistently higher for CFRP-reinforced columns compared to their steel-reinforced counterparts with identical transverse reinforcement configurations. For specimens with φ6@100 stirrups, C1-B (CFRP) recorded 780 kN, representing a 1.3% increase over C1-A (770 kN), while for φ8@100 stirrups, C3-B achieved 850 kN, a 5.6% increase over C3-A (805 kN).

This enhancement is primarily attributed to the effectiveness of confinement, which is influenced by both stirrup diameter and spacing. Increasing the stirrup diameter and reducing spacing enhance lateral restraint, delay crack initiation, and increase the effectively confined concrete core, as reported by Dhakal & Su^38^, and Mander et al.^39^. In addition, CFRP stirrups exhibit linear-elastic behavior, providing continuously increasing confining pressure with increasing strain, unlike steel stirrups, which tend to maintain nearly constant confinement after yielding^40,41^.

The influence of transverse reinforcement configuration was further confirmed, as increasing stirrup diameter from 6 mm to 8 mm (at 100 mm spacing) increased P_cr_ by 4.5% for steel columns and 9.0% for CFRP columns. In contrast, reducing spacing from 150 mm to 100 mm (at 6 mm diameter) resulted in smaller increases of 2.7% and 1.3% for steel and CFRP columns, respectively, indicating that stirrup diameter plays a more significant role in delaying crack initiation. The highest P_cr_ was achieved by specimen C3-B (φ8@100), with a 13.3% increase compared to the low confined specimen C2-A (steel, φ6@150). These findings are consistent with previous studies highlighting the importance of adequate transverse reinforcement in delaying cracking and improving confinement efficiency^42,43^. Higher cracking loads in CFRP-reinforced columns indicate an improved resistance to early damage, which can contribute to enhanced durability and reduced maintenance requirements, supporting ACI 440.1R-15 (2015)^44^, recommendations for proper transverse reinforcement detailing in FRP-reinforced concrete members.

**Table 9:** Test results for experimental columns.

Column ID	$\:\left({\boldsymbol{P}}_{\boldsymbol{c}\boldsymbol{r}}\right)$ (kN)	$\:{(\mathbf{P}}_{\boldsymbol{U}})$ (kN)	$\:{(\boldsymbol{\Delta\:}}_{\boldsymbol{U}})$ (mm)	(DF)	(E) (kN.mm)
C1 -A (STφ 6@100) -EXP.	770	925	5.48	1.0584	2440
C2 -A (STφ 6@150) -EXP.	750	910	5.585	1.0474	2429
C3 -A (STφ 8@100) -EXP.	805	1181.4	5.86	1.0668	3129
C4 -A (STφ 8@150) -EXP.	790	1050.1	5.74	1.0627	2800
C1 -B (CFRP φ6@100) -EXP.	780	917.75	5.9	1.0678	3250
C2 -B (CFRP φ6@150) -EXP.	770	835.31	5.33	1.0507	2282
C3 -B (CFRP φ8@100) -EXP.	850	1084.88	5.86	1.0751	3142
C4 -B (CFRP φ8@150) -EXP.	825	1007.07	5.69	1.0721	2984

***P***_cr_ refers to the cracking load, ***P***_u_ signifies the ultimate load, Δu indicates the displacement at ultimate load (***δ***_u_), and ***DF*** and ***E*** represent the ductility factor and energy absorption, respectively.

**Fig. 9:**
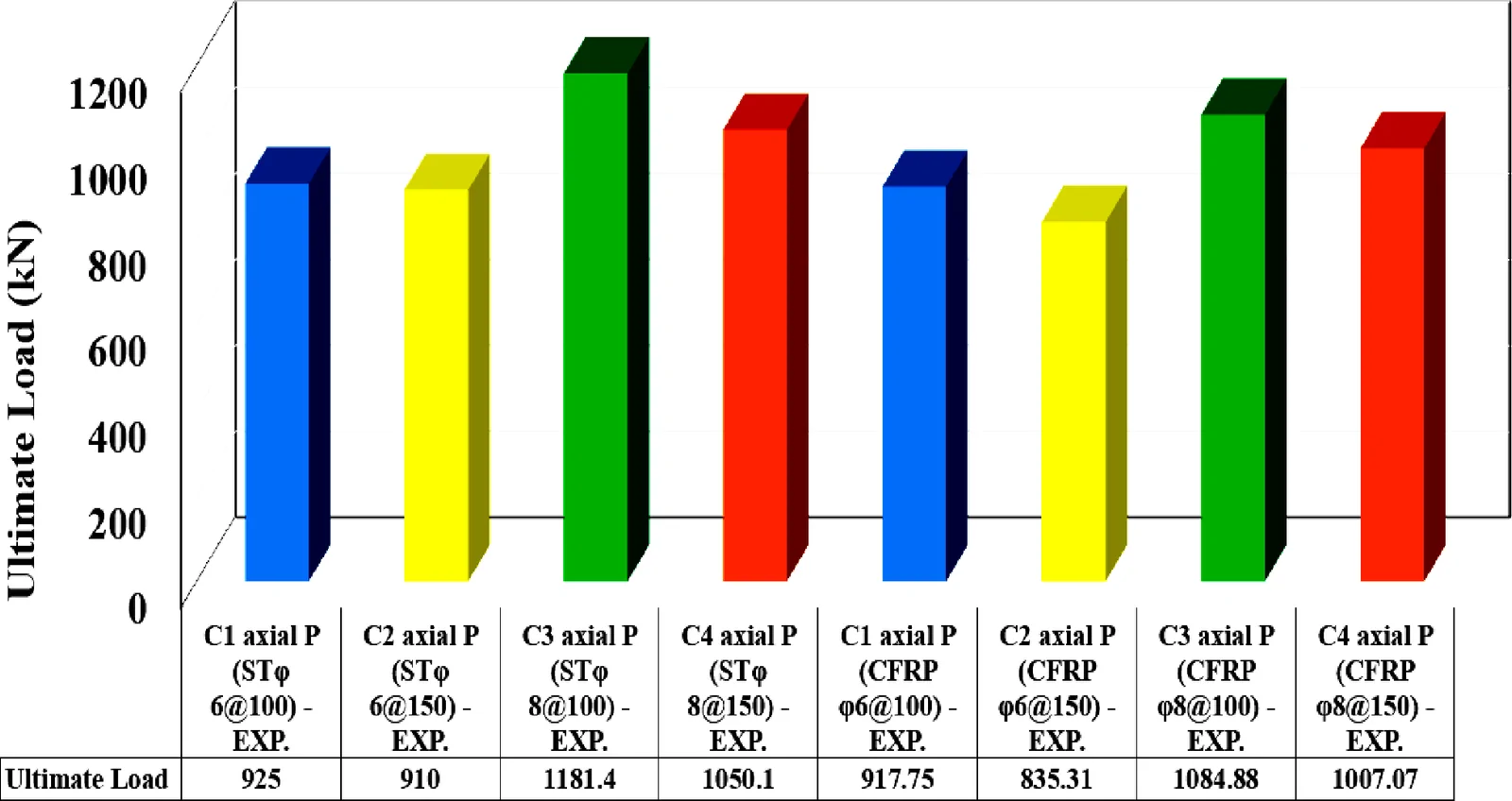
Ultimate load of tested columns.

According to Table 9; Fig. 9, the ultimate axial load capacity (Pu) showed that steel-reinforced columns generally achieved slightly higher ultimate loads than their CFRP counterparts. Specimen C3-A (steel, φ8@100) recorded the highest load at 1181.4 kN, while C3-B (CFRP) reached 1084.88 kN (8.2% reduction). In contrast, the difference was minimal for lightly confined specimens, where C1-A reached 925 kN compared to 917.75 kN for C1-B (only 0.8% reduction). Overall, CFRP columns achieved between 91.8% and 99.2% of the capacity of steel-reinforced columns when adequate confinement was provided.

This difference can be explained by the distinct mechanical behavior of the reinforcement materials. Steel reinforcement exhibits ductile behavior characterized by yielding followed by strain hardening, allowing stresses to approach its full yield strength^39^. In contrast, CFRP reinforcement remains linear elastic up to failure, without a yielding plateau. Consequently, the stress developed in CFRP bars is limited by the ultimate compressive strain of concrete (≈ 0.002–0.003) and the elastic modulus (≈ 112 GPa), resulting in lower attainable stress levels compared to conventional steel reinforcement^26,28^.

Despite this limitation, the results demonstrate that CFRP bars can effectively serve as longitudinal reinforcement in HPC columns when combined with adequate confinement. Properly confined CFRP-reinforced columns, particularly with φ8@100 transverse reinforcement, can achieve performance levels comparable to steel-reinforced columns, making them a viable alternative in applications where corrosion resistance is critical^5,28,44^.

### Failure mode

**Fig. 10:**
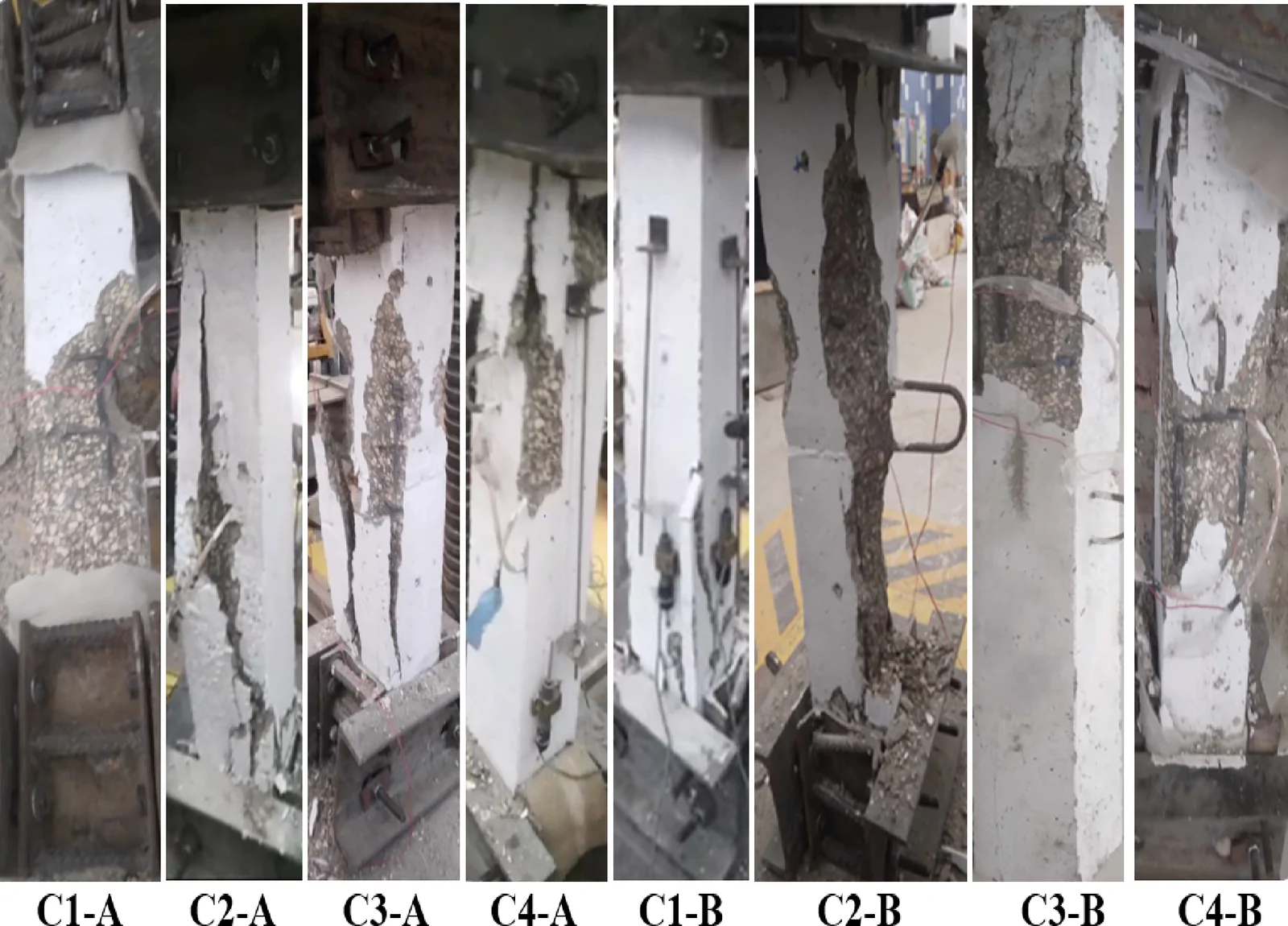
Failure mode for all column tested specimens.

Based on the failure modes illustrated in Fig. 10, specimens reinforced with φ6@150 stirrups (C2-A and C2-B) demonstrated lower performance, characterized by the formation of a wide diagonal crack followed by sudden brittle failure, which can be attributed to insufficient confinement^28^. In contrast, specimens reinforced with φ8@100 stirrups (C3-A and C3-B) exhibited a more ductile response, with failure governed by gradual concrete crushing. This improvement is associated with the larger stirrup diameter and reduced spacing, which enhanced core confinement effectiveness^45^.

A comparison between the two groups (A&B) shows that steel-reinforced specimens (Group A) developed fewer but wider cracks prior to failure, ultimately leading to a relatively brittle collapse. Meanwhile, CFRP-reinforced specimens (Group B) exhibited visible cracking but tended to fail suddenly under low confinement conditions, reflecting the linear-elastic nature of CFRP^27^. Among all specimens, C3-B (CFRP, φ8@100) demonstrated the most favorable behavior, with a gradual crushing mode and no evidence of diagonal failure. Conversely, C2-A (steel, φ6@150) and C2-B (CFRP, φ6@150) showed the least desirable performance, both failing in a brittle manner accompanied by diagonal concrete spalling^28,45^.

Although CFRP bars are primarily recognized for their tensile performance, they also contributed to the axial load-carrying capacity of the tested columns through their compressive stiffness and compatibility with the surrounding concrete. Despite their lower compressive strength compared with steel reinforcement, the CFRP-reinforced columns achieved comparable ultimate loads when adequate transverse confinement was provided. This indicates that the overall behavior was governed primarily by concrete confinement rather than by the compressive strength of the longitudinal reinforcement alone.

### Load-axial displacement (deflection) behavior

The axial displacement values presented represent the net axial shortening measured using LVDTs. Minor fluctuations observed near the origin of some load–displacement curves (e.g., specimens C1-A and C2-A) are attributed to seating and alignment effects during the initial loading stage rather than indicating a reversal of the axial displacement direction. Therefore, all recorded axial displacements correspond to positive axial shortening throughout the tests.

**Fig. 11:**
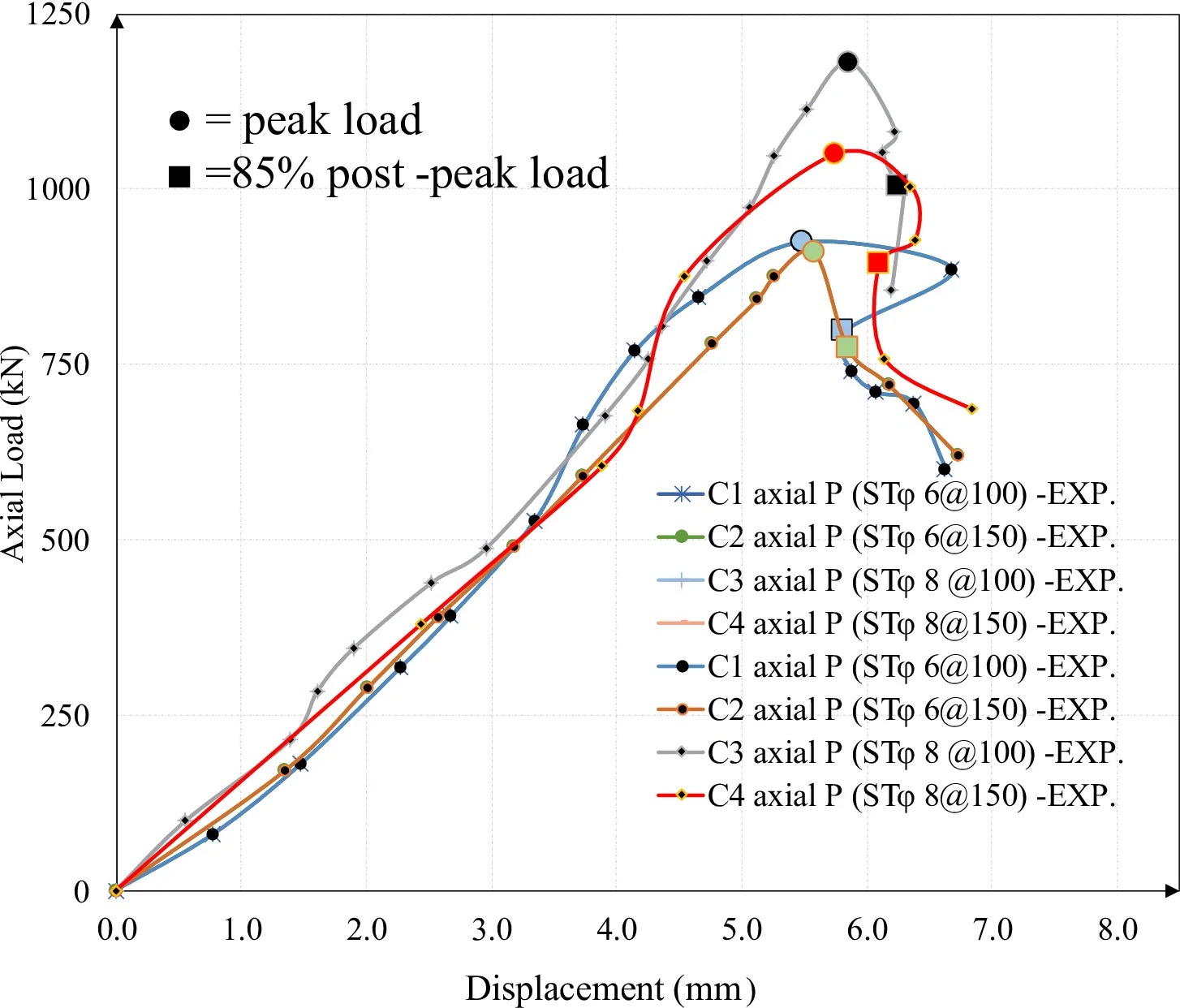
Effect of stirrups spacing and diameters on the load-axial displacement curves for columns reinforced by Steel bars (longitudinal bar and stirrups).

**Fig. 12:**
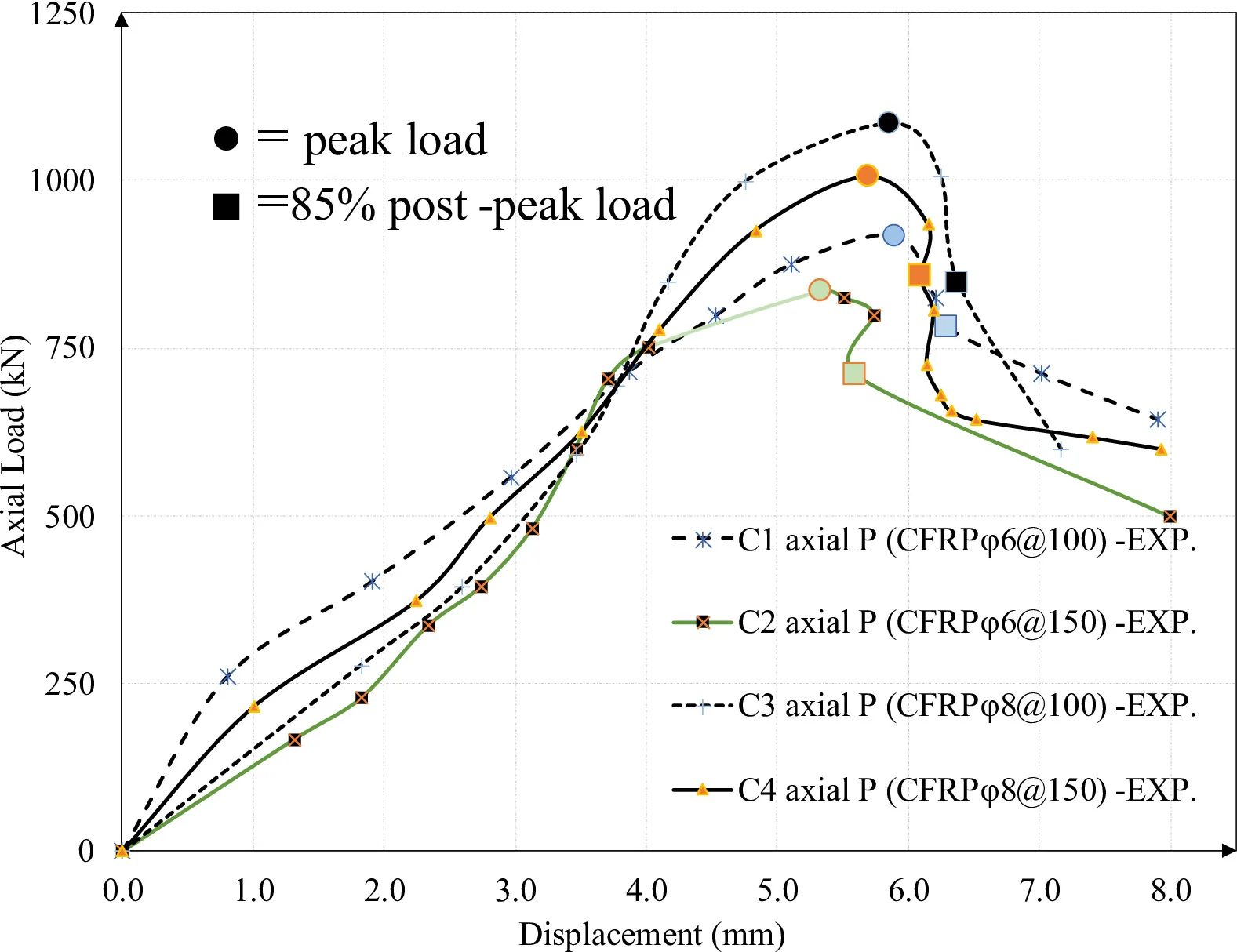
Effect of stirrups spacing and diameters on the load-axial displacement curves for columns reinforced by CFRP bars (longitudinal bar and stirrups).

**Fig. 13:**
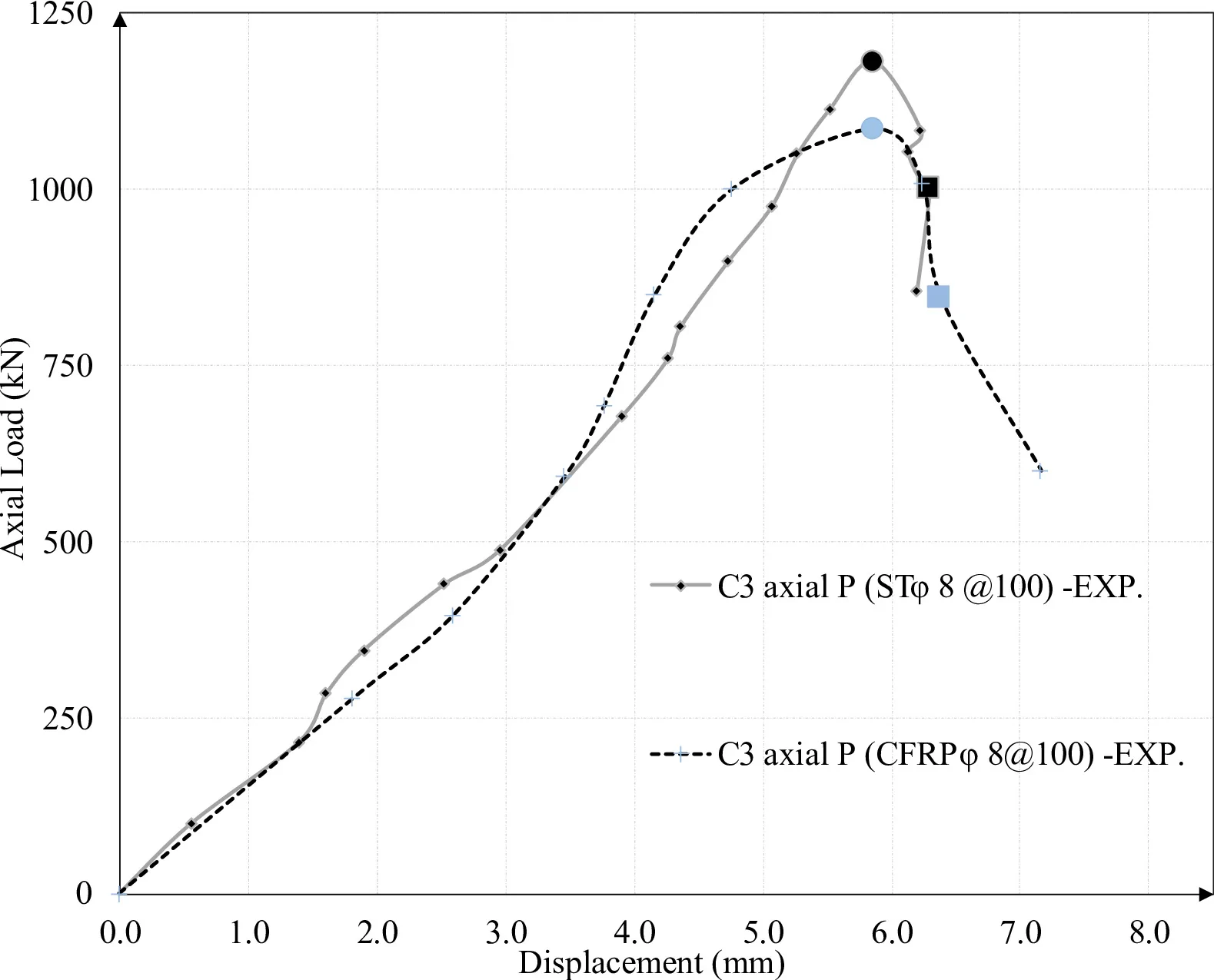
Effect of columns reinforced by steel versus CFRP bars (longitudinal bar and stirrups) on the load-axial displacement curves.

The load–axial displacement data were acquired at a sampling frequency of 10 Hz, resulting in approximately 500–800 data points per test. The peak load (Pu) and the corresponding 85% post-peak load (0.85Pu), used in the ductility index calculation, are indicated in Figs. 11, 12 and 13. The curves exhibit an initial linear elastic behavior followed by a nonlinear region approaching ultimate capacity, which is characteristic of confined concrete columns^39^. For steel-reinforced columns shown in Fig. 11, the effect of stirrup spacing on both ultimate load and displacement is clearly observed. Specimen C2-A (φ6@150) recorded an ultimate load of 910 kN with a displacement of 5.585 mm, while C1-A (φ6@100) achieved a higher ultimate load of 925 kN with a displacement of 5.48 mm. At the peak load of C2-A (910 kN), C1-A exhibited a displacement of 5.20 mm, which is 6.9% lower than that of C2-A. This indicates that reducing stirrup spacing from 150 mm to 100 mm enhances confinement effectiveness, leading to increased load capacity and reduced axial deformation under the same load level. These results agree with the findings of Dhakal and Su (2018)^38^, who reported that reducing stirrup spacing enhances confinement efficiency, as well as with Mander et al. (1988)^39^, who showed that properly confined concrete can achieve higher strength and reduced axial strains. A comparable trend was also observed for specimens with larger stirrup diameters (φ8@100 and φ8@150), further highlighting that both stirrup spacing and diameter significantly influence the load–displacement response of steel-reinforced HPC columns.

For CFRP-reinforced columns presented in Fig. 12, the load-displacement behavior also shows the beneficial effect of closer spacing, but with distinct characteristics compared to steel specimens. Specimen C2-B (φ6@150) recorded 800 kN with a displacement of 5.25 mm, while C1-B (φ6@100) achieved the same load with a lower displacement of 4.65 mm, representing a 11.43% reduction in displacement due to closer spacing. For the φ8 group, C4-B (φ8@150) at a load of 1000 kN recorded a displacement of 5.69 mm, whereas C3-B (φ8@100) achieved the same load with a displacement of only 4.76 mm, which is a 20.13% reduction. This significant difference highlights that closer spacing (100 mm) combined with larger diameter (φ8) in CFRP columns provides superior confinement efficiency compared to wider spacing (150 mm).

However, HPC columns reinforced with CFRP exhibited smaller axial displacements at comparable load levels, indicating greater stiffness and enhanced confinement effectiveness provided by CFRP stirrups as for example presented in Fig. 13 steel specimen C3-A (φ8@100) recorded 970 kN with a displacement of 5.07 mm, while CFRP specimen C3-B (φ8@100) achieved the same load with a lower displacement of 4.60 mm, representing a 9.27% reduction in displacement. This observation agrees with Wang et al. (2023)^45^, who reported that CFRP confinement improves ductility while effectively controlling deformation, as well as with Hadi et al. (2018)^46^, who reported that FRP stirrups reduced axial displacements in columns reinforced with FRP stirrups compared to those with steel stirrups under similar loading conditions.

### Column’s ductility (DF)

Ductility is defined as the ability of a structural element to undergo significant deformation without a substantial reduction in load-carrying capacity after reaching its peak load^39,47^. It is commonly quantified using the ductility index (DI), expressed as DI = δ₀.₈₅/δ_u_, where δ_u_ is the displacement corresponding to the ultimate (peak) load, and $\:{\delta\:}_{{}^{0}{.}^{85}}$is the displacement on the post-peak descending branch at which the load decreases to 85% of the ultimate load ($\:{P}_{u}$). For specimens exhibiting sudden or brittle failure, where the load dropped rapidly after reaching the peak, $\:{\delta\:}_{{}^{0}{.}^{85}}$ was taken as the displacement corresponding to the first recorded data point at which the applied load fell below $\:0.85{P}_{u}$. Higher DI values indicate greater deformation capacity and ductility^42,45^.

**Fig. 14:**
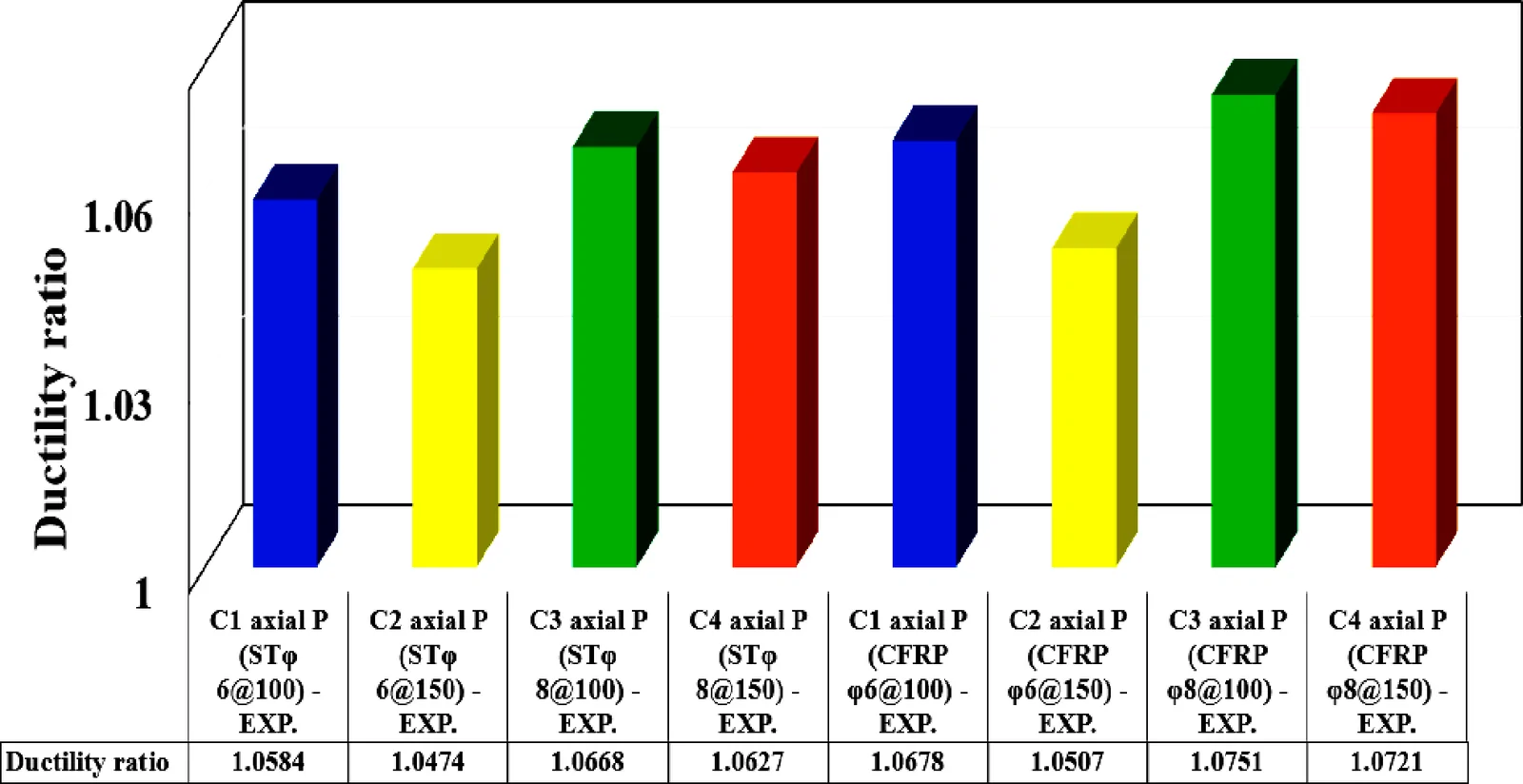
Ductility ratio of tested columns.

In the current study, as illustrated in Figs. 11 and 12, and 14, CFRP-reinforced columns recorded slightly higher ductility indices than their steel counterparts, with increases ranging from 0.3% to 0.9%. Among all specimens, C3-B (CFRP, φ8@100) exhibited the highest DI of 1.0751, which is about 0.8% higher than its steel counterpart C3-A (1.0668). A similar trend was observed for specimen C1-B, which reached a DI of 1.0678, reflecting a 0.9% increase over C1-A (1.0584). This improvement can be linked to the more effective confinement provided by CFRP stirrups, which helps limit lateral expansion, delays the onset of concrete crushing, and allows the column to sustain larger deformations^5,28^. On the other hand, the lowest ductility among the CFRP-reinforced columns was recorded for C2-B (φ6@150), with a DI of 1.0507, likely due to the reduced confinement associated with the wider spacing and smaller stirrup diameter^42^.

Consistent with the findings of Wang et al. (2023)^45^, increasing the level of confinement significantly enhances the ductility index. Their study reported that specimens confined with five layers of CFRP achieved DI values of 1.66 and 1.33, compared to 1.00 and 1.08 for those with only two layers. Similarly, in the current investigation, improving confinement from φ6@150 to φ8@100 increased ductility by 2.33% for CFRP columns (from 1.0507 to 1.0751) and by 1.85% for steel columns (from 1.0474 to 1.0668). This demonstrates that CFRP columns derive greater benefit from enhanced confinement, attributable to their lower modulus of elasticity and linear-elastic behavior, which allow larger axial deformations when properly confined^28,40^.

Specimens with low confinement showed a sharp drop in post-peak load, with ductility index (DI) values close to 1.00, reflecting brittle behavior. In contrast, well-confined specimens exhibited a more gradual post-peak response, reflected in higher DI values^45^. Steel-reinforced columns generally developed smoother descending branches, mainly due to the yielding of the longitudinal bars. On the other hand, CFRP-reinforced columns were able to reach comparable, or even superior, ductility when adequate confinement was provided, as the confinement mechanism governs the response and compensates for the absence of yielding in CFRP longitudinal reinforcement^39,48^.

This behavior is evident in the 2.33% increase in ductility between the low confined specimen C2-B and the well-confined specimen C3-B, highlighting the critical role of transverse reinforcement design in promoting ductile behavior in FRP-reinforced high-performance concrete columns^28,43^.

### Energy absorption (E)

Energy absorption (E) is defined as the area under the load–displacement curve and depends on both the ultimate load and the corresponding ultimate displacement, serving as a key indicator of structural toughness^42,49,50^.

In the present study, the energy absorption was calculated as the area under the experimentally recorded load–displacement curve from the onset of loading ($\:\delta\:=0$) to the point of failure, defined as the displacement corresponding to either the first recorded load below 50% of the ultimate load ($\:{P}_{u}$) or the termination of the test, whichever occurred first. The area was evaluated using the trapezoidal numerical integration method.

**Fig. 15:**
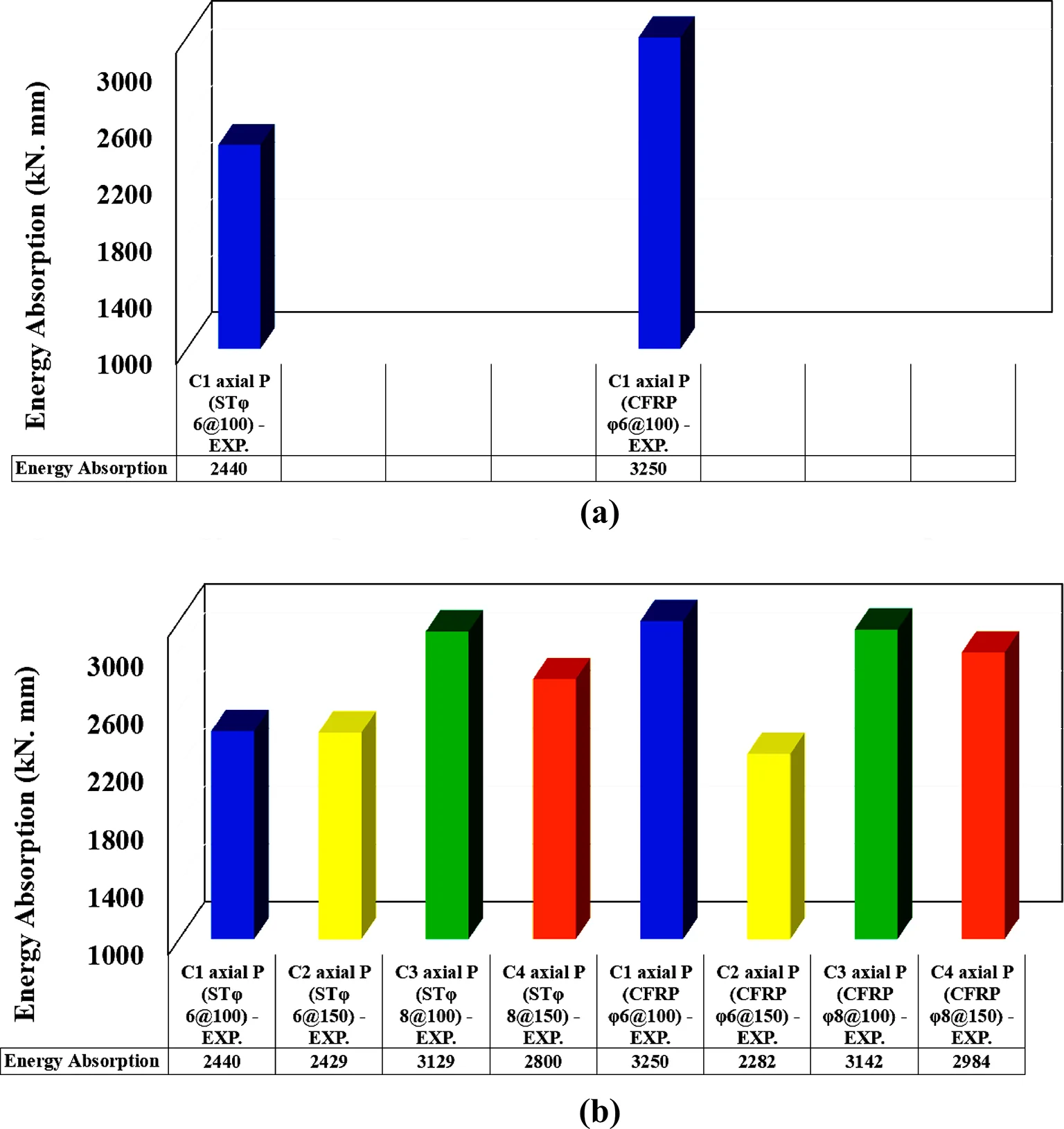
Energy Absorption Capacity (E) for (**a**) C1-A and C1-B Specimens, (**b**) Tested Specimens.

As illustrated in Fig. 15a, specimen C1-B (CFRP, φ6@100) achieved higher energy absorption of 3250 kN·mm, representing a 33.2% increase over its steel-reinforced counterpart C1-A (2440 kN·mm). This improvement demonstrates that adequate transverse confinement enables CFRP-reinforced columns to exploit their greater deformability and dissipate more energy prior to failure^5,40,50,57^. In contrast, specimen C2-B (CFRP, φ6@150) exhibited the lowest energy absorption, reaching only 2282 kN·mm, which is approximately 6.0% lower than its steel counterpart C2-A (2429 kN·mm) (Fig. 15b). This reduction reflects the effect of insufficient confinement, which promotes premature concrete crushing and limits the energy dissipation capacity^28,42^.

Although the load–displacement responses of specimens C3-A and C3-B (φ8@100) shown in Fig. 13 are very similar, their calculated energy absorption values were also nearly identical, reaching 3129 kN·mm and 3142 kN·mm, respectively, corresponding to a difference of only 0.4%. This indicates that under a high level of transverse confinement, CFRP reinforcement provides energy absorption comparable to that of steel reinforcement. Therefore, the most significant improvement in energy absorption was observed for the C1-B/C1-A specimen pair, as demonstrated by the results presented in Fig. 15b, rather than for the C3-B/C3-A pair.

Improving confinement from φ6@150 to φ8@100 increased the energy absorption by 37.69% for CFRP columns (from 2282 to 3142 kN·mm) and by 28.8% for steel columns (from 2429 to 3129 kN·mm), indicating that CFRP-reinforced columns benefited more from enhanced transverse confinement^28,40^. For specimens with φ8@100 stirrups, both reinforcement types exhibited nearly identical energy absorption capacities, with C3-B and C3-A recording 3142 and 3129 kN·mm, respectively (a difference of only 0.4%).

This demonstrates that under a high level of confinement, CFRP reinforcement can achieve energy dissipation comparable to that of conventional steel reinforcement. For specimens with φ8@150 stirrups, however, the CFRP-reinforced specimen exhibited a more pronounced improvement, achieving approximately 6.57% higher energy absorption than its steel counterpart (2984 versus 2800 kN·mm). These findings confirm that increasing the stirrup diameter enhances confinement effectiveness and enables CFRP-reinforced columns to develop improved energy dissipation even at wider stirrup spacing^38,50,57^.

### Strain profiles

#### Steel & CFRP—longitudinal bar strain profiles

**Fig. 16:**
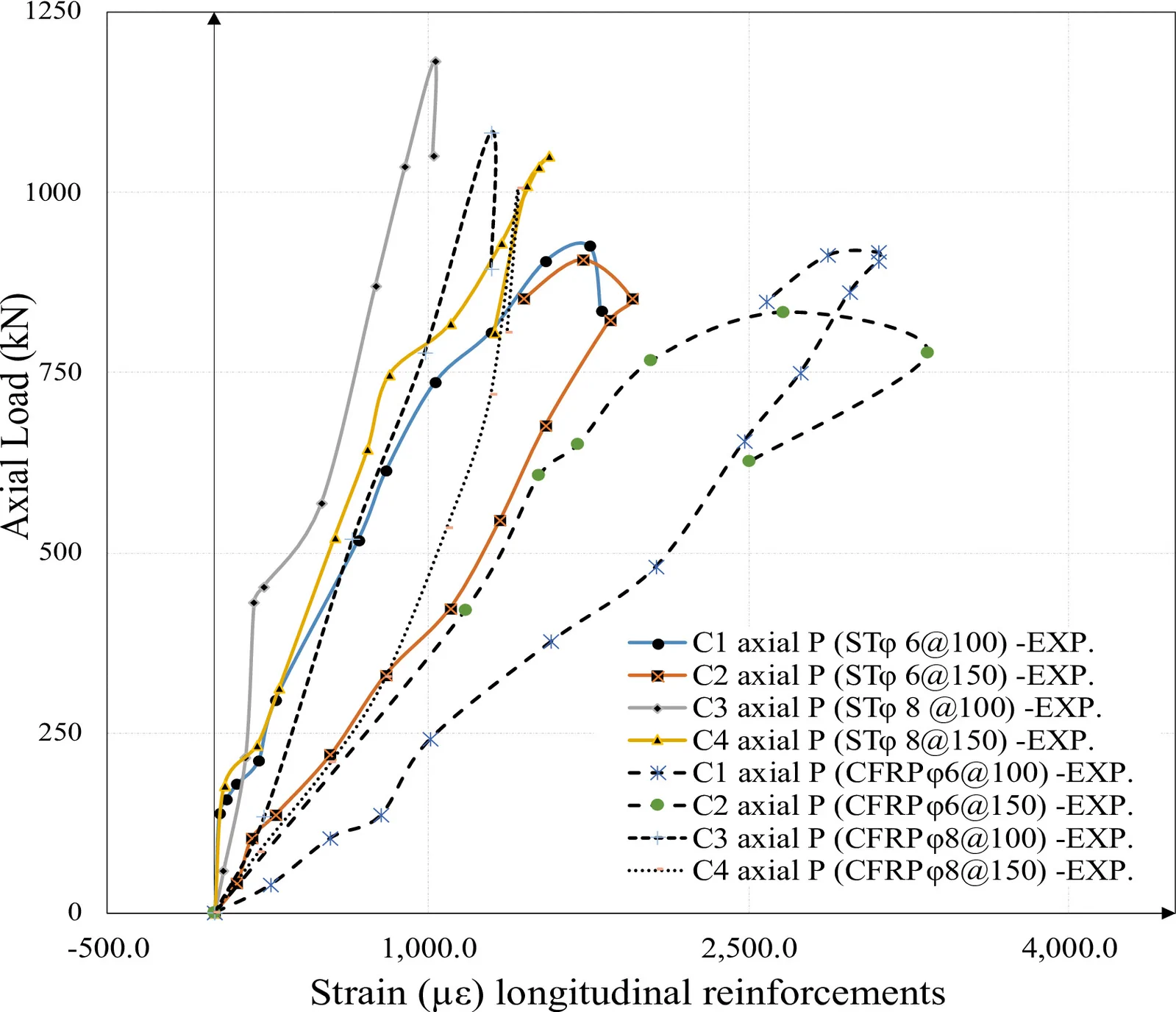
Axial Load versus outermost Longitudinal bar strain at mid-height for the CFRP and steel bars reinforced HPC columns.

Figure 16 illustrates the relation between the applied axial load and the strain recorded in the outermost longitudinal bars at mid-height for both steel-reinforced (Group A: C1-A to C4-A) and CFRP-reinforced (Group B: C1-B to C4-B) columns, highlighting the contribution of longitudinal reinforcement to axial load-carrying capacity and the interaction between the reinforcement and the surrounding concrete under progressive loading.

CFRP longitudinal bars displayed a distinctly different response characterized by linear-elastic behavior throughout the loading history with no evidence of yielding. At equivalent load levels, the CFRP bars exhibited higher strains than their steel counterparts, reflecting their lower modulus of elasticity. For example, at an axial load of approximately 1000 kN, specimen C3-B (CFRP, φ8@100) exhibited a strain of about 1200 µε, whereas its steel-reinforced counterpart, C3-A, recorded a strain of approximately 860 µε under the same loading condition. This corresponds to a 39.5% higher strain in the CFRP-reinforced specimen. This difference is attributed to the lower modulus of elasticity of CFRP bars (approximately 112 GPa) compared to that of steel (200 GPa), which results in greater axial deformation under identical loading^5,28,40^.

The maximum compressive strain recorded in the longitudinal CFRP bars during column testing was approximately 2600 μm/m (0.26%). Considering the ultimate tensile strain capacity of the CFRP bars obtained from the material tensile tests (4000 μm/m, Fig. 3), the maximum recorded strain corresponds to approximately 65% of the ultimate tensile strain capacity.

For both steel and CFRP groups, the effect of stirrup spacing and diameter was evident. Specimens with closer spacing (100 mm) and larger diameter (φ8) generally exhibited lower longitudinal bar strains at equivalent load levels compared to specimens with wider spacing (150 mm) and smaller diameter (φ6). This is because better transverse confinement reduces lateral expansion, which in turn reduces the axial strain demand on the longitudinal bars. For example, within the CFRP group at an axial load of 750 kN, specimen C3-B recorded a strain of 900 µε, whereas C4-B exhibited an estimated strain of approximately 1200 µε. This indicates a 33.3% lower strain in the specimen with closer stirrup spacing. This observation is consistent with the findings of Dhakal and Su (2018), who reported that reducing stirrup spacing enhances confinement effectiveness and limits the strain in longitudinal reinforcement^38^.

#### Steel & CFRP—stirrups or transverse bar strain profiles

**Fig. 17:**
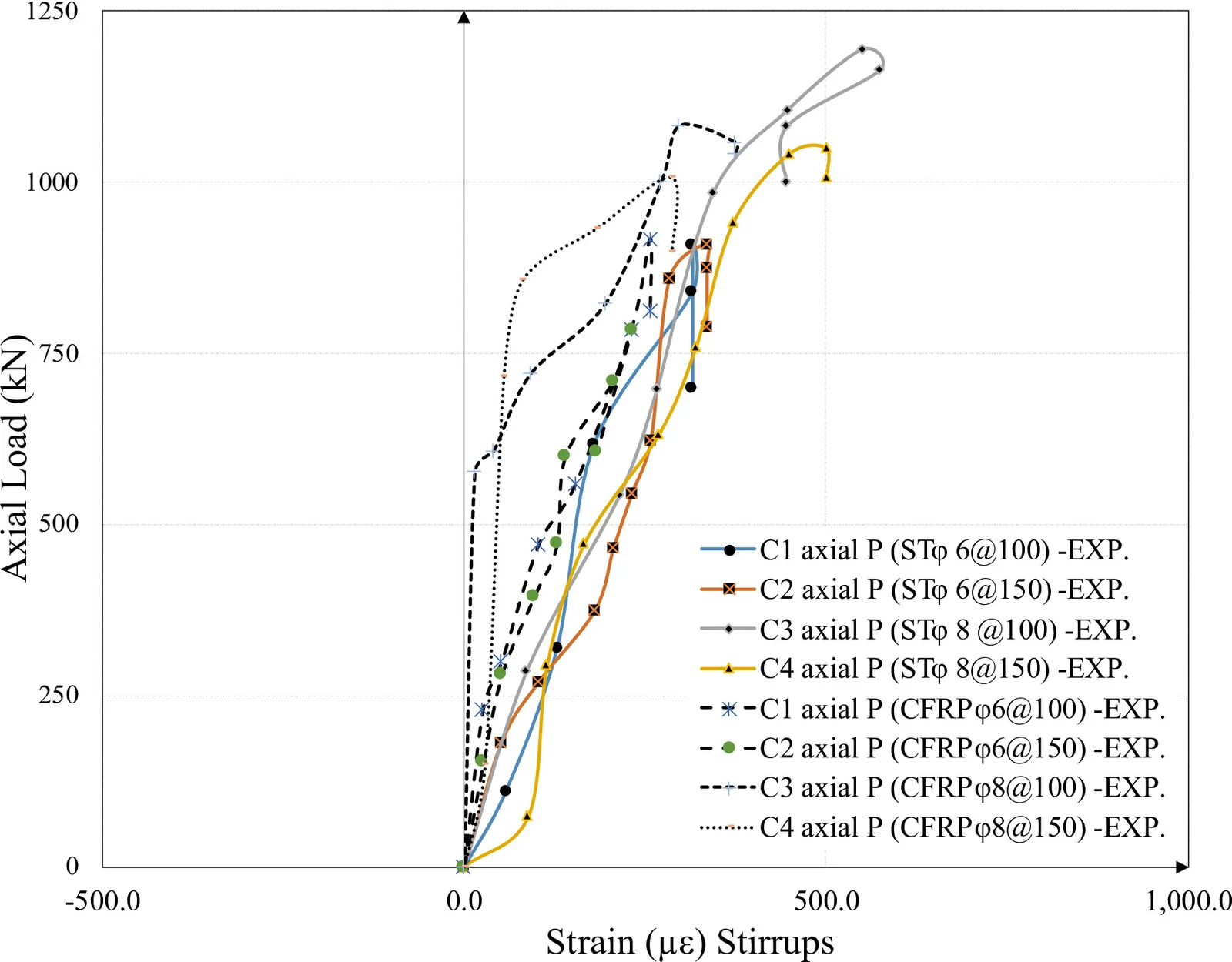
Axial Load versus outermost stirrups strain at mid-height of the studied HPC columns steel bars.

Figure 17 demonstrates the axial load versus strain measured in the outermost stirrups at mid-height for all tested specimens, elucidating the activation and effectiveness of transverse reinforcement in confining the concrete core throughout the loading history. The slope of each curve reflects confinement stiffness, defined as the gain in axial load per unit increase in transverse strain. This parameter is a key indicator of the effectiveness of the confining system, as a stiffer confinement provides greater lateral pressure for the same amount of concrete dilation, leading to more significant improvements in strength and ductility^51^.

For specimens reinforced with steel stirrups (C1-A to C4-A), the initial slope is relatively steep but gradually decreases as the steel begins to yield direction. As a result, transverse strain increases rapidly without a corresponding increase in load. This behavior is consistent with the findings of Lapshinov and Deminov (2018)^52^, who reported that reducing stirrup spacing leads to lower transverse strains and Poisson’s ratio, while improving the effective modulus of elasticity in compression members.

In contrast, specimens reinforced with CFRP stirrups (C1-B to C4-B) exhibit a steeper and more sustained slope. This reflects the linear elastic nature of CFRP, which does not yield and continues to effectively restrain lateral expansion until near failure. Similar observations were reported by Wang et al. (2023)^45^, who found that CFRP confinement significantly enhances both load-carrying capacity and ductility compared to conventional methods.

Regarding spacing, specimens with 100 mm stirrup spacing (C1-A and C3-A for steel; C1-B and C3-B for CFRP) show steeper slopes than those with 150 mm spacing (C2-A and C4-A for steel; C2-B and C4-B for CFRP), indicating improved confinement with closer spacing. This trend agrees with Hadi et al. (2018)^46^, who demonstrated that reducing stirrup spacing leads to higher ultimate axial strain.

Furthermore, increasing stirrup diameter from φ6 to φ8 produces steeper slopes for both material types, with the steepest slope observed for C3-B (CFRP, φ8@100). Even at 150 mm spacing, CFRP specimens such as C2-B and C4-B produce a steeper slope than steel specimens at 100 mm spacing (C1-A and C3-A), indicating that material type has a stronger influence on confinement stiffness than spacing alone. After peak load, the slope becomes negative for all specimens, but the descending branch is steeper for steel specimens (C1-A to C4-A), indicating more brittle failure, while CFRP specimens (C1-B to C4-B) show more gradual load drops, consistent with the finding that CFRP confinement significantly improves post-peak ductility^45^.

#### Axial strain profiles of concrete

**Fig. 18:**
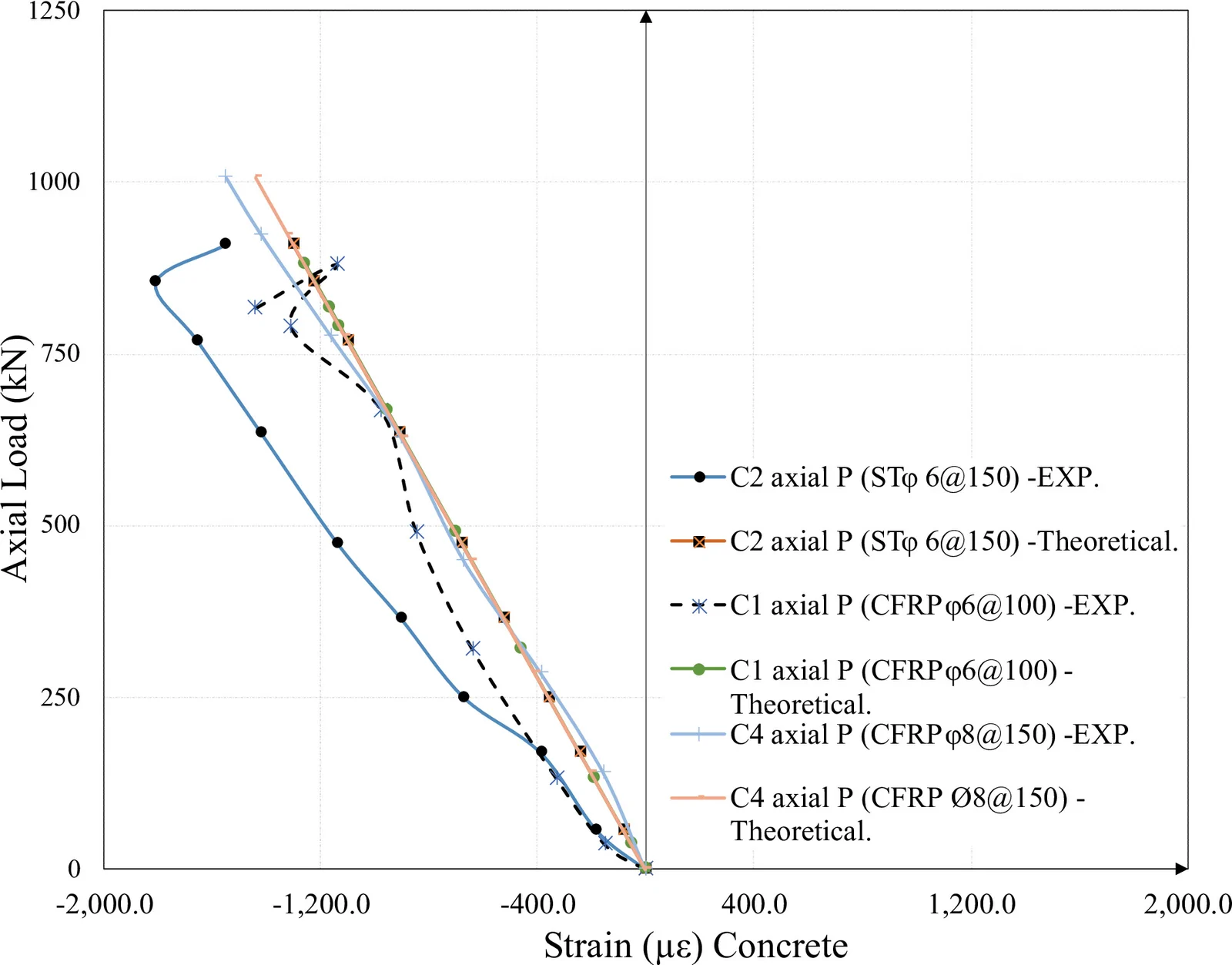
Axial Load versus outermost concrete strain at mid-height for the CFRP and steel bars reinforced HPC columns.

Figure 18 presents the relationship between the applied axial load and the axial strain measured on the concrete surface at mid-height for selected specimens, including steel-reinforced (C2-A) and CFRP-reinforced (C1-B and C4-B) columns, along with their corresponding theoretical predictions. The theoretical axial strain was calculated according to ESS 203 (2020), which specifies the modulus of elasticity for concrete as E_c_ = 4400 √ f_cu_ = 4400 × √50 = 31,112 MPa^33^. The cross-sectional area of all columns was A_g_ = 150 × 150 = 22,500 mm². The axial stress was computed as σ = P/A_g_, and the theoretical axial strain was computed as ε_th_ = σ/E_c_ = P/(E_c_ × A_g_). For example, at a load of 750 kN, the theoretical stress was σ = 750,000 N/22,500 mm² = 33.33 MPa, and the theoretical strain was ε_th_ = 33.33/31,112 = 0.001071 = 1071 µε.

For specimen C2-A (steel, φ6@150), the experimental strain at 750 kN was − 1600 µε, representing a 33.1% difference from the theoretical prediction, while at 855 kN, the experimental strain was − 1800 µε compared to a theoretical value of −1221 µε (32.2% difference). This consistent underestimation indicates that steel stirrups at 150 mm spacing provide minimal confinement for high-strength concrete, as previously reported by Mander et al. (1988)^39^, who reported that lightly confined concrete develops much higher axial strains than those predicted by elastic theory, as a result of lateral expansion.

But for specimen C1-B (CFRP, φ6@100), the experimental strain at 750 kN was − 1200 µε compared to the theoretical value of −1071 µε (10.8% difference), and at 817 kN, the experimental strain was − 1440 µε compared to −1167 µε (19.0% difference). The slight underestimation by the theoretical values reflects the improved confinement provided by the CFRP wrap and closer stirrup spacing, which delays reduces axial strain. This conservative prediction is desirable for design purposes, as noted by Abed et al. (2024)^5^, and El-Sayed et al. (2023)^28^, who demonstrated that linear elastic models can reliably and conservatively estimate axial deformation in CFRP-reinforced concrete columns.

Also, specimen C4-B (CFRP, φ8@150), the theoretical value showed excellent agreement with experimental results. At 750 kN, the experimental strain was − 1100 µε compared to −1071 µε (2.6% difference), and at 1007 kN, the experimental strain was − 1550 µε compared to −1439 µε (7.2% difference). This close agreement validates the ability of the theoretical prediction to account for the effect of stirrup configuration on axial strain behavior, and it confirms that larger stirrup diameter (8 mm) enhances confinement. Dhakal and Su (2018)^38^, similarly reported that increasing transverse reinforcement diameter improves confinement efficiency by providing greater lateral stiffness, while Wang et al. (2023)^45^ demonstrated that CFRP wrapping combined with adequate internal stirrups significantly reduces axial strain and delays failure in high-performance concrete columns.

## Theoretical capacity calculation and comparison with experimental results

### Theoretical capacity calculation

To estimate the axial load capacity of the tested columns, a set of equations was adopted. These equations were selected to reflect different assumptions regarding the contribution of longitudinal reinforcement, particularly in the case of CFRP bars, where the compressive behavior is less certain compared to steel.

For steel-reinforced columns, the nominal axial capacity was first calculated using Eq. 1 (ACI 318 − 19)^53^, which is widely accepted for conventional reinforced concrete design. This equation assumes full contribution of both concrete and steel reinforcement and serves as a baseline reference for evaluating the accuracy of other equations. The cylinder compressive strength (fc’) was derived from the cube compressive strength (fcu = 50.2 MPa) using fc’ = 0.8 × fcu = 40.16 MPa, consistent with ACI 318 − 19 (Table 19) and ECP 203–2018^54^. The nominal axial capacity can be expressed as:

1 $$\:\begin{array}{cccc}&\:{P}_{n}=0.85{f}_{c}^{{\prime\:}}\left({A}_{g}-{A}_{s}\right)+{A}_{s}{f}_{y}\end{array}$$

$$\:{P}_{n}=0.85\times\:40.16\times\:\left(22500-452.4\right)+\left(452.4\times\:420\right)=\mathrm{942,258}{\hspace{0.17em}}\mathrm{N}\approx\:\mathrm{942}\:\mathrm{kN}$$

To provide a conservative comparison, the axial capacity was also calculated using the ECP 203–2018^54^, formulation (Eq. 2), which adopts reduced coefficients for both concrete and steel contributions. This equation is known for its conservative predictions and is included to assess the lower-bound capacity estimates.

Although the experimentally measured yield strength of the 12 mm longitudinal steel bars was (f_y_ = 435) MPa, a nominal yield strength of (f_y_ = 420) MPa was adopted in the theoretical calculations, corresponding to Grade 42/60 specified by the supplier and consistent with ESS 203/2020^33^.

2 $$\:\begin{array}{cccc}&\:{P}_{n}=0.35{f}_{cu}\times\:\left({A}_{c}-{A}_{sc}\right)+0.67{f}_{y}{A}_{sc}\end{array}$$

$$\:{P}_{n}=0.35\times\:50.2\times\:\left(22500-452.4\right)+0.67\times\:420\times\:452.4\approx\:\mathrm{515}\:\mathrm{kN}$$

Where $\:{f}_{c}^{{\prime\:}}$ is the cylinder compressive strength of concrete, $\:{A}_{g}$ is the gross area of the section, $\:{A}_{s}$ and $\:{A}_{sc}$ are the area of longitudinal steel bars, $\:{f}_{y}$ is the yield stress of steel reinforcement, $\:{f}_{cu}$ is the cubic compressive strength of the concrete.

For CFRP-reinforced columns, different approaches were considered due to the uncertainty associated with the compressive behavior of FRP materials. According to ACI 440.1R-15 (Eq. 3)^44^, the contribution of FRP bars in compression is neglected, leading to:

3 $$\:\begin{array}{cccc}&\:{P}_{n}=0.85{f}_{c}^{{\prime\:}}\left({A}_{g}-{A}_{f}\right)\end{array}$$

$$\:{P}_{n}=0.85\times\:40.16\times\:\left(22500-452.4\right)\approx\:\mathrm{753}\:\mathrm{kN}$$

This assumption provides a conservative estimate and represents the minimum expected capacity.

To achieve a more realistic prediction, the equation proposed by Zrar et al. (2019)^7^, (Eq. 4) was adopted. This equation accounts for the contribution of CFRP bars through a strain-based approach, assuming linear elastic behavior up to a limiting strain. The axial capacity is calculated as:

4 $$\:\begin{array}{cccc}&\:{P}_{n}={{\rm\:Y}}{f}_{c}^{{\prime\:}}\left({A}_{g}-{A}_{f}\right)+0.003\left(K{E}_{f}{A}_{f}\right)\end{array}$$

$$\:{P}_{n}=\mathrm{752,250}+0.003\times\:\left(0.8\times\:\mathrm{111,300}\times\:452.4\right)\approx\:\mathrm{874}\:\mathrm{kN}$$

Where $\:{{\rm\:Y}}$ = 0.85 is the concrete stress block coefficient (Zrar et al., 2019)^7^, * K* = 0.8 for CFRP bars, and $\:{E}_{f}$ is the elastic modulus of FRP bars.

Finally, the equation proposed by Maranan et al. (2016); (Eq. 5)^55^, was used as a refined formulation, incorporating a higher concrete contribution and a reduced strain limit for FRP reinforcement:

5 $$\:\begin{array}{cccc}&\:{P}_{n}=0.9{f}_{c}^{{\prime\:}}\left({A}_{g}-{A}_{f}\right)+0.002{E}_{FRP}{A}_{FRP}\end{array}$$

$$\:{P}_{n}=0.9\times\:40.16\times\:22047.6+0.002\times\:\mathrm{111,300}\times\:452.4\approx\:\mathrm{897}\:\mathrm{kN}$$

Where $\:{E}_{FRP}$is the elastic modulus of the CFRP bars and $\:{A}_{f}$is the area of the longitudinal CFRP bars. Equation (5) was developed through regression analysis of an extensive experimental database to achieve close agreement with the reported experimental results^50^.

In the present study, the strain developed in the CFRP bars was assumed to be governed by strain compatibility with the surrounding concrete. Accordingly, the limiting CFRP compressive strain of 0.002 adopted in Eq. (5) was selected based on: (i) strain compatibility with concrete, whose ultimate compressive strain is typically taken as 0.002–0.003 in ACI 318 − 19; (ii) the findings of Maranan et al. (2016)^55^, who demonstrated that limiting the CFRP compressive strain to 0.002 provides good agreement with experimental results; and (iii) the experimental observations of Afifi et al. (2014b), who reported CFRP compressive strains of approximately 0.002–0.0025 prior to failure^7,26,50^.

To account for the contribution of transverse confinement, a preliminary confinement modification was applied to all theoretical prediction models using Eq. (6). The proposed modification increases the predicted load capacity in proportion to the volumetric transverse reinforcement ratio, thereby enabling the models to distinguish between specimens with different stirrup diameters and spacings. As expected, the improvement is more evident for models that already provide reasonable estimates of the basic axial capacity^7,55^, whereas the improvement remains limited for highly conservative models such as ECP 203–2018 because the primary source of underestimation originates from the basic formulation itself rather than from neglecting the confinement effect^54^.

The form of Eq. (6) is inspired by classical confinement models^39,56^, in which the increase in concrete compressive strength is related to the confining pressure provided by transverse reinforcement. The proposed modification extends this concept to the column axial capacity by introducing a confinement efficiency coefficient)**α**(.

α calibrated from the experimental data of the present study.

6 $$\:{P}_{n,conf}={P}_{n,basic}\left(1+\:\boldsymbol{\upalpha\:}{\:{\rho\:}_{s}f}_{c}^{{\prime\:}}\right)$$

Where *ρ*_s_ is the volumetric transverse reinforcement ratio, and α is a confinement efficiency coefficient calibrated from experimental data. This is presented as a recommendation for future research rather than a fully validated model.

### Comparison with experimental results

A consistent trend observed across all specimens is that the experimental capacities generally exceed the theoretical predictions. This behavior is reflected in the $\:\frac{{P}_{exp}}{{P}_{theo}}$ ratios, which are predominantly greater than unity. This systematic underestimation suggests that the adopted analytical models, while safe for design purposes, do not fully capture the actual load-carrying mechanisms of the tested columns. In particular, the beneficial effects of confinement appear to be underestimated or entirely neglected in most formulations.

**Fig. 19:**
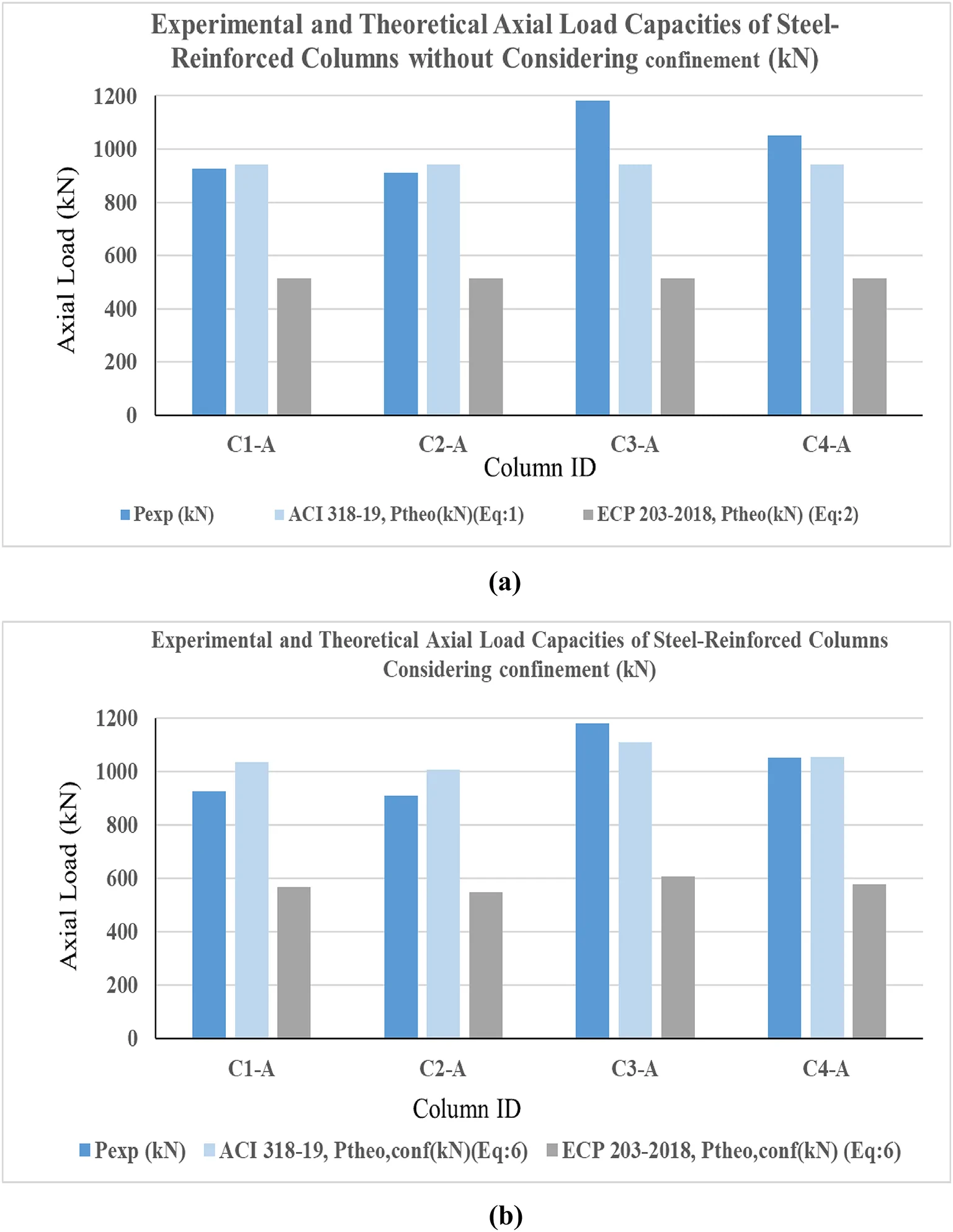
Experimental and Theoretical Axial Load Capacities of Steel-Reinforced Columns (**a**) without Considering Confinement, (**b**) Considering Confinement.

**Fig. 20:**
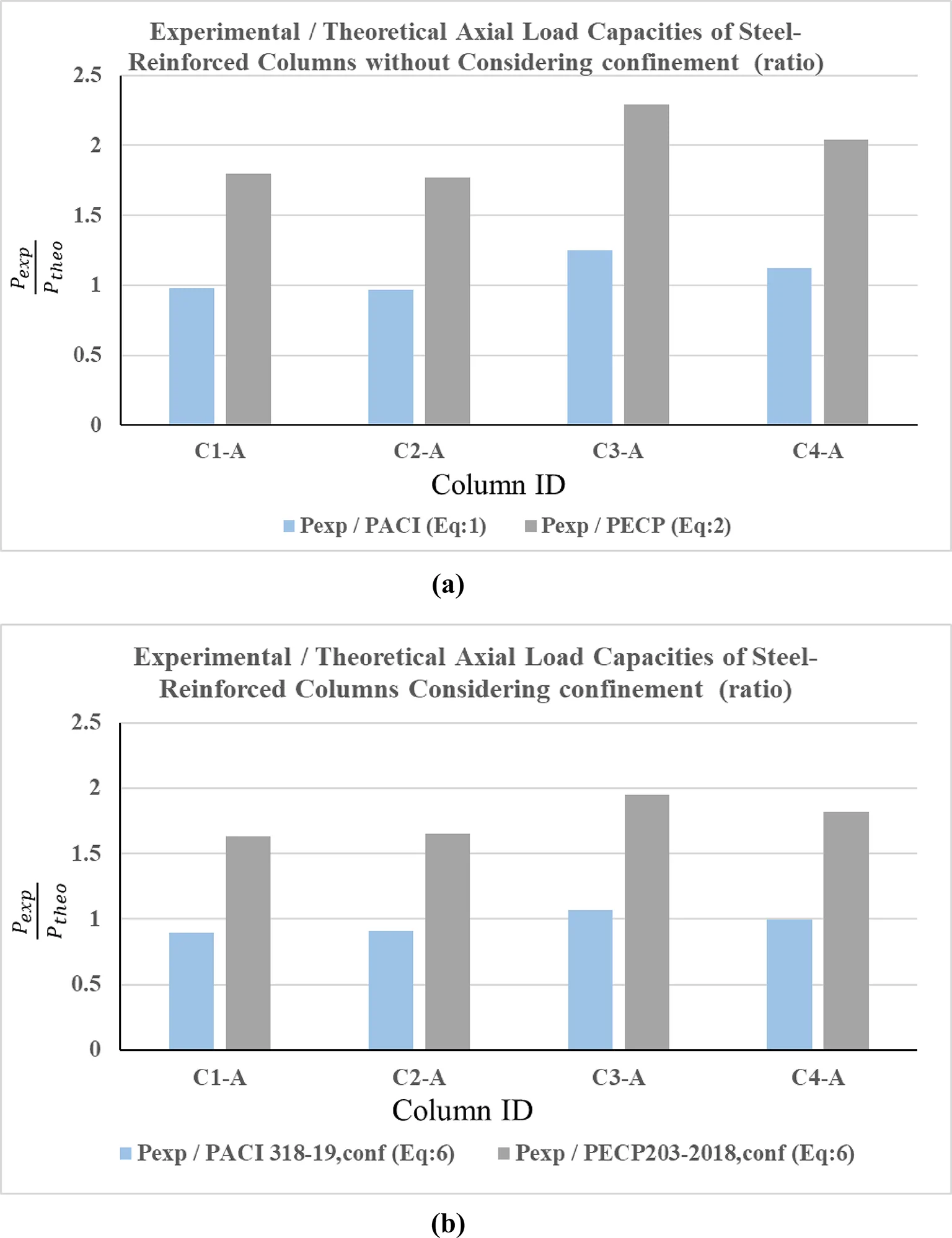
Experimental/Theoretical Axial Load Capacities of Steel-Reinforced Columns (**a**) without Considering Confinement (ratio), (**b**) Considering Confinement (ratio).

For steel-reinforced columns (group A), as shown in Table 10; Figs. 19a and 20a (without Considering Confinement), the ACI 318 − 19 equation shows good agreement with the experimental results, with ratios ranging from 0.97 to 1.25^53^. The higher ratios obtained for C3-A and C4-A, particularly specimen C3-A, are attributed to the enhanced confinement provided by the larger stirrup diameter (φ8) and closer spacing (100 mm), which resulted in a higher transverse reinforcement ratio. This improved confinement delayed crack propagation, enhanced the confinement of the concrete core, and consequently increased the axial load-carrying capacity of the columns beyond that predicted by the ACI equation. These findings suggest that although the ACI 318 − 19 equation provides satisfactory and generally conservative predictions, it does not fully account for the increased confinement effectiveness associated with heavily confined HPC columns^53^.

When the confinement effect is considered, as shown in Table 11; Figs. 19b and 20b, the theoretical predictions exhibit a clearer differentiation according to the transverse reinforcement configuration. The confinement-modified ACI 318 − 19 predictions increase from 1005 kN for C2-A (φ6@150) to 1110 kN for C3-A (φ8@100), reflecting the increase in the volumetric transverse reinforcement ratio. Consequently, the corresponding (P_exp_/P_ACI, conf_) ratios range from 0.89 to 1.06, compared with 0.97–1.25 when confinement was not considered. In particular, the predicted capacity for C3-A increases from 942 kN to 1110 kN, resulting in a ratio of 1.06 compared with 1.25 for the basic ACI 318 − 19 prediction^53^. Similarly, for C4-A (φ8@150), the confinement-modified prediction reaches 1053 kN, which is in close agreement with the experimental capacity of 1050.10 kN, giving a ratio of approximately 1.00. These results indicate that incorporating the confinement effect improves the ability of the ACI 318 − 19 formulation to capture the experimentally observed increase in axial load-carrying capacity associated with larger-diameter and/or more closely spaced stirrups^53^.

For the ECP 203–2018 formulation, the confinement modification increases the predicted capacities from 515 kN to 550–607 kN, depending on the transverse reinforcement configuration. However, the resulting (P_exp_/P_ECP, conf_) ratios remain relatively high (1.63–1.95), indicating that the ECP 203–2018 formulation remains substantially conservative even after considering confinement^54^. This suggests that, although explicitly incorporating the transverse reinforcement ratio improves the representation of confinement effects, it cannot fully compensate for the inherent conservatism of the basic ECP formulation. Overall, the results demonstrate that introducing a confinement-dependent term enables the theoretical models to distinguish between specimens with different stirrup diameters and spacings, particularly for the more confined specimens C3-A and C4-A.

**Table 10:** Comparison Between Experimental and Theoretical Results for Steel-Reinforced Columns without Considering Confinement.

Column ID	*P*_exp_ (kN)	ACI 318 − 19 *P*_theo_ (kN) (Eq:1)	*P*_exp_/*P*_ACI_ (Eq:1)	ECP 203–2018 *P*_theo_ (kN) (Eq:2)	*P*_exp_/*P*_ECP_ (Eq:2)
C1-A	925.00	942	0.98	515	1.80
C2-A	910.00	942	0.97	515	1.77
C3-A	1181.40	942	1.25	515	2.29
C4-A	1050.10	942	1.12	515	2.04

**Table 11:** Comparison Between Experimental and Theoretical Results for Steel-Reinforced Columns Considering Confinement.

Column ID	*P*_exp_ (kN)	ACI 318 − 19, *P*_theo_(kN) (Eq:1)	*P*_ACI_ _318−19, conf_ (Eq:6)	*P*_exp_ /*P*_ACI,318−19,conf_	ECP 203–2018, *P*_theo_(kN) (Eq:2)	*P*_ECP 203−2018, conf_ (Eq:6)	*P*_exp_ / *P*_ECP 203−2018, conf_
C1-A	925.00	942	1036	0.89	515	567	1.63
C2-A	910.00	942	1005	0.91	515	550	1.65
C3-A	1181.40	942	1110	1.06	515	607	1.95
C4-A	1050.10	942	1053	0.997	515	576	1.82

**Fig. 21:**
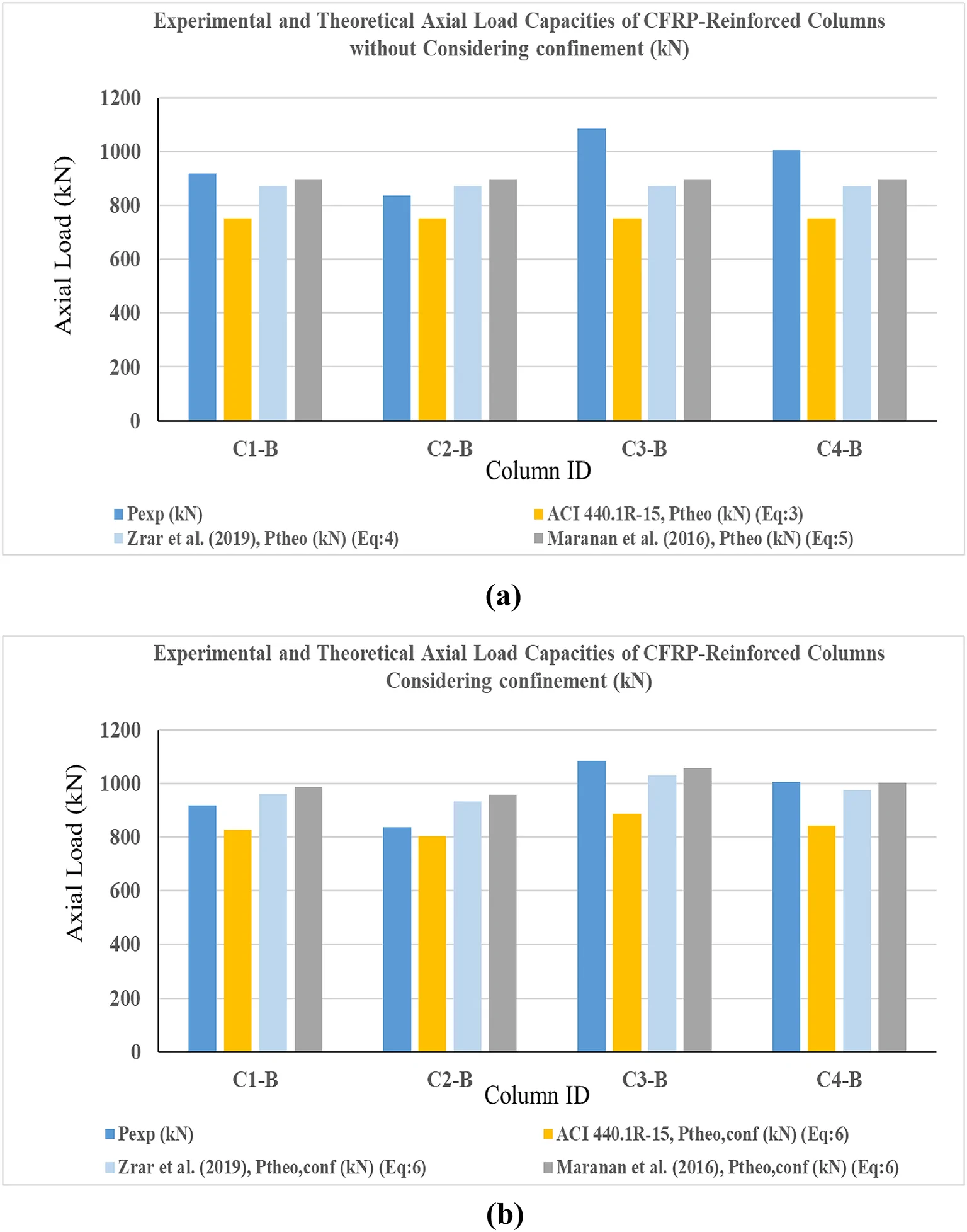
Experimental and Theoretical Axial Load Capacities of CFRP-Reinforced Columns (**a**) without Considering Confinement (kN), (**b**) Considering Confinement (kN).

**Fig. 22:**
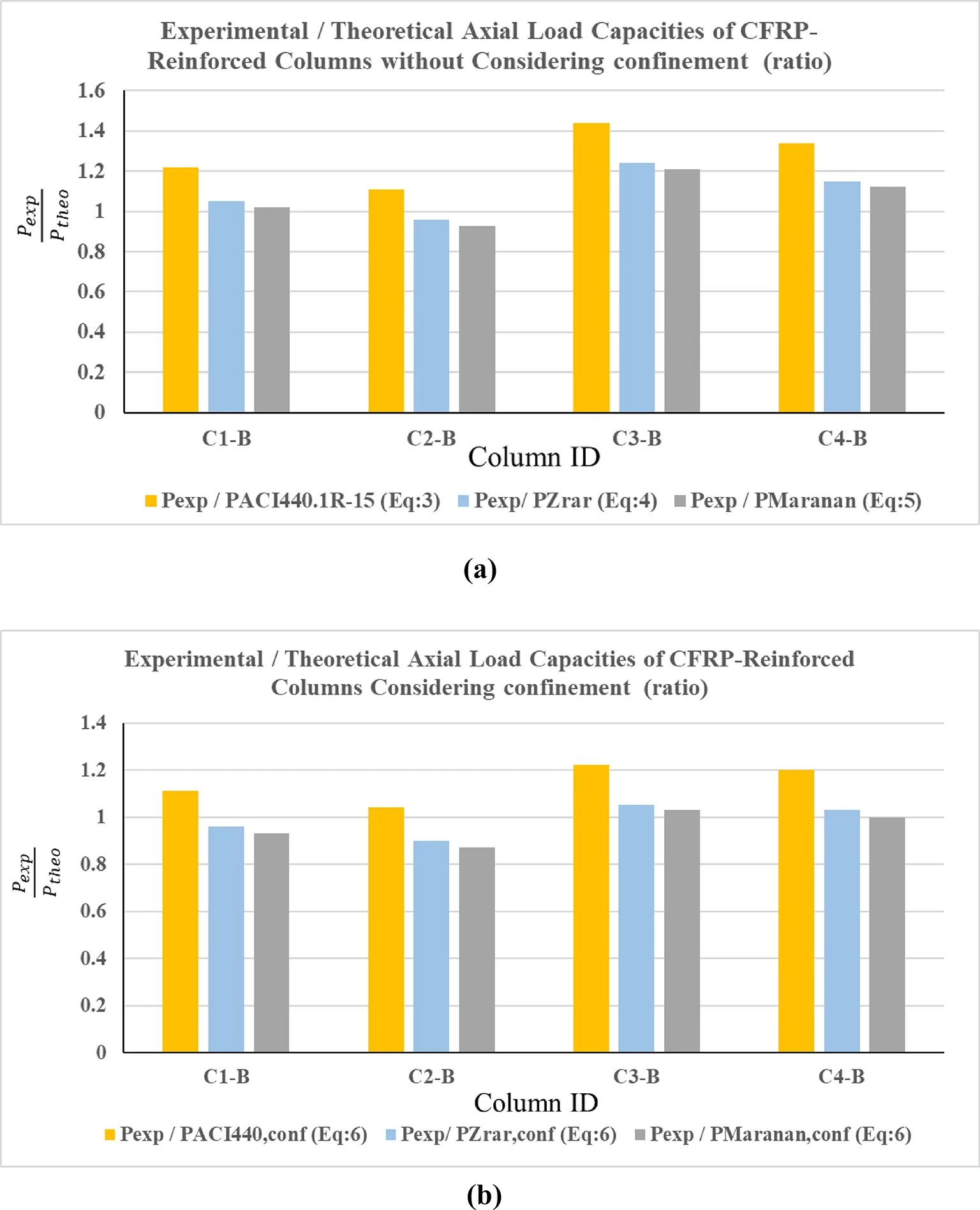
Experimental/Theoretical Axial Load Capacities of CFRP-Reinforced Columns (**a**) without Considering Confinement (ratio), (**b**) Considering Confinement (ratio).

For CFRP-reinforced columns (group B), as shown in Table 12; Figs. 21a and 22a, both strain-based models provide closer estimates to the experimental results. The Zrar et al. (2019)^7^ equation yields ratios between 0.96 and 1.24, while the Maranan et al. (2016)^55^ equation gives slightly lower values ranging from 0.93 to 1.21. The improved agreement reflects the importance of including the contribution of CFRP reinforcement in the analysis. Nevertheless, the higher ratios observed for C3-B and C4-B, particularly specimen C3-B, indicate that the confinement provided by the φ8 CFRP ties at 100 mm spacing significantly enhanced the load-carrying capacity beyond that predicted by the available models. Since the equations proposed by Zrar et al. (2019)^7^ and Maranan et al. (2016)^55^ primarily consider the contribution of longitudinal CFRP reinforcement and do not explicitly incorporate the effectiveness of transverse confinement, they tend to underestimate the capacity of well-confined specimens.

When the confinement effect is considered, as shown in Table 13; Figs. 21b and 22b, the theoretical predictions exhibit a clearer response to the different transverse reinforcement configurations. For the Zrar et al. (2019) model, the confinement-modified (P_exp_/P_Zrar, conf_) ratios range from 0.90 to 1.05, compared with 0.96–1.24 without considering confinement^7^. The closest agreement is obtained for C3-B (φ8@100), where the predicted capacity increases to 1030 kN compared with the experimental value of 1084.9 kN, giving a ratio of 1.05. Similarly, the Maranan et al. (2016)^55^ model provides confinement-modified ratios ranging from 0.87 to 1.03, with the prediction for C3-B increasing to 1058 kN and approaching the experimental capacity of 1084.9 kN. For C4-B (φ8@150), the modified predictions of 961 kN and 988 kN for the Zrar and Maranan models^7,55^, respectively, also demonstrate the influence of the transverse reinforcement configuration on the predicted capacity. In contrast, the ACI 440.1R-15 predictions remain relatively conservative after incorporating the confinement effect, with (P_exp_/P_ACI440,conf_) ratios ranging from 1.04 to 1.22^44^. Although the proposed modification increases the predicted capacity from 753 kN to 804–887 kN, the model still underestimates the experimental capacities, particularly for the more heavily loaded specimens C3-B and C4-B. Overall, these results indicate that explicitly incorporating the transverse reinforcement ratio improves the ability of the theoretical models, particularly the Zrar et al. (2019) and Maranan et al. (2016) formulations^7,55^, to reflect the experimentally observed influence of confinement. The improvement is particularly evident for specimens with larger-diameter and/or more closely spaced CFRP ties, confirming that the confinement contribution should be considered when predicting the axial capacity of CFRP-reinforced HPC columns.

**Table 12:** Comparison Between Experimental and Theoretical Results for CFRP-Reinforced Columns without Considering Confinement.

Column ID	*P*_exp_ (kN)	ACI440.1R-15, *P*_theo_ (kN) (Eq:3)	*P*_exp_/*P*_ACI440.1R−15,_ (Eq:3)	Zrar et al. (2019), *P*_theo_ (kN) (Eq:4)	*P*_exp_ / *P*_Zrar_(Eq:4)	Maranan et al. (2016), *P*_theo_ (kN) (Eq:5)	*P*_exp_ /*P*_Maranan_(Eq:5)
C1-B	917.75	753	1.22	874	1.05	898	1.02
C2-B	835.31	753	1.11	874	0.96	898	0.93
C3-B	1084.9	753	1.44	874	1.24	898	1.21
C4-B	1007.1	753	1.34	874	1.15	898	1.12

**Table 13:** Comparison Between Experimental and Theoretical Results for CFRP-Reinforced Columns Considering Confinement.

Column ID	*P*_exp_ (kN)	ACI 440.1R-15 (kN) Basic (Eq:3)	*P*_ACI_ _440, conf_ (Eq:6)	*P*_exp_/*P*_ACI 440, conf_	Zrar et al. (2019), *P*_theo_ (kN) basic (Eq:4)	*P*_Zrar, conf_ (Eq:6)	*P*_exp_ /*P*_Zrar_ _,conf_	Maranan et al. (2016), *P*_theo_ basic (kN) (Eq:5)	*P*_Maranan, conf_ (Eq:6)	*P*_exp_ /*P*_Maranan_,_conf_
C1-B	917.75	753	828	1.11	874	961	0.96	898	988	0.93
C2-B	835.31	753	804	1.04	874	932	0.90	898	958	0.87
C3-B	1084.9	753	887	1.22	874	1030	1.05	898	1058	1.03
C4-B	1007.1	753	828	1.11	874	961	0.96	898	988	0.93

### Discussion of model performance

A significant limitation of the theoretical models examined is that none of the basic formulations explicitly account for the influence of stirrup diameter, spacing, or transverse reinforcement ratio. The experimental capacity of steel-reinforced columns varies from 910 kN (C2-A, φ6@150) to 1181 kN (C3-A, φ8@100), corresponding to an increase of approximately 30%, while the ACI 318 − 19^53^, prediction remains constant at 942 kN. Similarly, CFRP-reinforced columns vary from 835 kN to 1085 kN, also corresponding to an increase of approximately 30%, while the Zrar et al. (2019) and Maranan et al. (2016) formulations^7,55^, provide constant predictions. This indicates that the basic theoretical formulations do not adequately capture the contribution of transverse reinforcement confinement, particularly for heavily confined HPC columns.

To address this limitation, the confinement-dependent modification proposed in Eq. (6) was applied to the basic theoretical formulations. The resulting predictions, presented in Tables 11 and 13; Figs. 19b, 20b and 21b, and 22b, demonstrate that incorporating the transverse reinforcement ratio enables the theoretical models to better reflect the experimentally observed variation in axial capacity. The improvement is particularly evident for the ACI 318 − 19 formulation for steel-reinforced columns and for the Zrar et al. (2019) and Maranan et al. (2016) formulations^7,55^ for CFRP-reinforced columns. This trend is consistent with the experimentally observed increase in axial capacity associated with larger stirrup diameters and/or closer spacing, which enhance confinement of the concrete core.

Nevertheless, the confinement modification does not eliminate the limitations of the underlying formulations. In particular, the ECP 203–2018 model remains substantially conservative even after considering confinement, indicating that its discrepancy is associated not only with the absence of an explicit confinement parameter but also with the inherent conservatism of its basic formulation^54^.

The basic ECP predictions correspond to experimental-to-predicted capacity ratios of approximately 1.77 to 2.29, while the confinement-modified predictions remain considerably below the experimental capacities. Thus, incorporating confinement alone is insufficient to overcome the conservatism inherent in the ECP formulation.

A modification factor (**λ**_*HPC*_) for the ECP 203–2018 formulation can be derived from the experimental-to-predicted capacity ratios:

7 $$\:{\boldsymbol{\lambda\:}}_{HPC}=\frac{{\boldsymbol{P}}_{\boldsymbol{e}\boldsymbol{x}\boldsymbol{p}.}}{{\boldsymbol{P}}_{\boldsymbol{E}\boldsymbol{C}\boldsymbol{P}}}=1.8\:to\:2.3$$

Accordingly, the modified ECP 203–2018 equation for HPC columns can.

be expressed as:

8 $$\:\begin{array}{cccc}&\:{\boldsymbol{P}}_{\boldsymbol{n},\boldsymbol{m}\boldsymbol{o}\boldsymbol{d}\boldsymbol{i}\boldsymbol{f}\boldsymbol{i}\boldsymbol{e}\boldsymbol{d}}={\boldsymbol{\lambda\:}}_{\boldsymbol{H}\boldsymbol{P}\boldsymbol{C}}\text{}\times\:\left[0.35{f}_{cu}\times\:\left({A}_{c}-{A}_{sc}\right)+0.67{f}_{y}{A}_{sc}\right]\:\end{array}$$

This suggests that for HPC columns with adequate confinement, the ECP 203–2018^54^, prediction may be conservatively increased by a factor of approximately 1.8 to 2.0 for design optimization purposes. The large discrepancy arises from three primary factors: (a) the ECP 203–2018 formulation employs intentionally conservative coefficients (0.35 for concrete and 0.67 for steel) calibrated for ordinary concrete (≤ 40 MPa); (b) the formulation does not account for the enhanced strength of confined concrete, which is particularly significant in HPC; and (c) the confined core of HPC columns develops higher stresses than the code allows, resulting in substantial over-conservatism. However, further experimental validation across a wider range of HPC compressive strengths and confinement levels is required before proposing code modifications.

Overall, the findings confirm that explicitly considering transverse reinforcement is important for representing the confinement-dependent axial capacity of HPC columns. The proposed confinement-dependent modification provides a preliminary analytical means of accounting for this effect and improves the ability of several existing formulations to distinguish between different confinement levels.

However, it should not be regarded as a validated design equation. A broader experimental database covering different concrete strengths, longitudinal and transverse reinforcement ratios, stirrup configurations, and confinement levels is required for independent calibration and validation before the proposed modification can be recommended for general design applications.

## Conclusions

This study investigated the axial compressive behavior of HPC columns reinforced with steel and CFRP bars, with varying stirrup diameters (6 and 8 mm) and spacings (100 and 150 mm). Based on experimental and theoretical results, the following conclusions are drawn:

- Steel columns achieved higher ultimate loads than CFRP columns (0.8–8.2% reduction). Well-confined CFRP columns achieved about 92–99% of steel capacity, confirming CFRP bars effectively sustain compression under adequate confinement.
- CFRP‑reinforced HPC columns showed slightly lower ultimate loads but higher displacements. At the same axial load of 970 kN, the CFRP specimen C3‑B achieved a displacement of 4.60 mm, representing a 9.27% reduction relative to the 5.07 mm displacement observed for the steel specimen C3‑A.
- Enhancing the confinement level from φ6@150 to φ8@100 produced a 2.33% rise in ductility for CFRP columns, exceeding the 1.85% increase observed in steel columns.
- Higher confined CFRP columns achieved superior energy absorption for example C1-B achieved 3250 kN·mm, comparable to C1-A (2440 kN·mm), confirming inadequate confinement limits deformation capacity.
- The results obtained from all CFRP-reinforced columns collectively indicate that the accuracy of theoretical predictions is highly dependent on confinement efficiency.
- Incorporating a preliminary confinement-dependent modification improved the ability of several theoretical formulations to reflect the experimentally observed confinement effect, particularly the ACI 318 − 19 formulation for steel-reinforced columns and the Zrar et al. (2019) and Maranan et al. (2016) formulations for CFRP-reinforced columns. However, the ECP 203–2018 formulation remained substantially conservative even after considering confinement.
- The experimental results indicate that the confinement level is the governing parameter controlling the structural response of CFRP-reinforced HPC columns. When adequate confinement was provided (φ8@100), the reduction in axial capacity relative to steel-reinforced columns became marginal, demonstrating that confinement efficiency has a greater influence on column performance than the type of longitudinal reinforcement.
- Based on the tested specimens, CFRP-reinforced HPC columns exhibited structural performance comparable to steel-reinforced columns when adequate confinement was provided. These findings suggest that CFRP reinforcement is a promising alternative for short HPC columns under concentric axial compression.
- A modification factor (**λ**_*HPC*_ = 1.8 to 2.3) was derived from the experimental-to-predicted capacity ratios for the ECP 203–2018 formulation. For design optimization of HPC columns with adequate confinement, a conservative value of **λ**_*HPC*_ ≈ 1.8 to 2.0 is recommended. This modification increases the ECP 203–2018 prediction from approximately 515 kN to 927–1030 kN, bringing it closer to the experimental range of 910–1181 kN. However, further experimental validation across a wider range of HPC compressive strengths and confinement levels is required before proposing code modifications.

## Limitations and future research directions

The present study is limited by the number of specimens tested and the range of investigated parameters. Although the experimental results provide valuable insight into the behavior of CFRP-reinforced HPC columns under concentric axial compression, additional experimental investigations involving a larger number of specimens and broader parameter ranges are required before more general design recommendations can be established.

Accordingly, the following experimental research areas should be considered in future studies to allow a more comprehensive investigation of the observed properties:

1. Examine the effect of closely spaced CFRP stirrups (with spacing less than 100 mm) on the structural performance of ultra-high-performance concrete (UHPC) columns reinforced with CFRP longitudinal bars under axial compression.
2. Assess the influence of axial load eccentricity on the structural response of high-performance concrete (HPC) and UHPC columns reinforced with CFRP bars as a replacement for conventional steel longitudinal reinforcement.
3. Compare the behavior of normal-strength and high-strength concrete columns reinforced with carbon fiber bars when subjected to fire exposure under sustained loading, including specimens reinforced with carbon fiber bars.
4. Investigate the effects of elevated temperatures on the mechanical performance of carbon fiber bars under applied load.
5. Develop and independently validate confinement-dependent analytical models using a broader experimental database covering different concrete strengths, longitudinal and transverse reinforcement ratios, stirrup configurations, and confinement levels.

## Data Availability

All data generated or analyzed during this study are included in this published article.
